# Consumers’ Food Safety Expectations and Risk Perceptions of Produce From Small and Medium‐Sized Farms

**DOI:** 10.1111/1750-3841.70527

**Published:** 2025-09-23

**Authors:** Juan Carlos Archila‐Godínez, Claudia Kotanko, Renee Wiatt, Maria I. Marshall, Yaohua Feng

**Affiliations:** ^1^ Department of Food Science Purdue University West Lafayette Indiana USA; ^2^ Department of Agricultural Economics Purdue University West Lafayette Indiana USA

**Keywords:** consumer survey, food safety label, food quality, farm size, produce safety

## Abstract

Food safety concerns associated with fresh produce have gained prominence due to recurring foodborne outbreaks and recalls. However, consumer awareness and perceptions regarding food safety in small and medium‐sized farm (SMF) operations remain underexplored. This study assessed consumer expectations and risk perceptions of produce from SMFs through a survey of 916 U.S. consumers. While 85% of respondents considered food safety a minimum quality standard, it was often viewed as secondary to attributes such as freshness, quality, and local production. Most higher‐income consumers (63%) opposed the exemption of SMFs from the Food Safety Modernization Act's Produce Safety Rule. Consumers identified farmers as key actors in ensuring produce safety, with 75% rating them as extremely influential in maintaining food safety. Additionally, 47% of respondents attributed responsibility to farmers when presented with a hypothetical foodborne outbreak scenario, while a majority believed all commercially sold food should be safe regardless of farm size. Structured equation modeling revealed the interrelationships among constructs, including demographic characteristics, produce handling practices, food safety knowledge, expectation of food safety as a minimum quality standard, perceptions of produce from SMFs, and perceptions of food safety standards for SMFs. These findings provide insight into consumer attitudes toward produce safety and regulatory expectations for SMFs. The results suggest that consumers expect food safety to be an inherent characteristic of fresh produce, yet their perceptions of risk and responsibility are shaped by broader considerations of food quality, sourcing, and trust in agricultural producers.

## 1 Introduction

In the United States, most farms (96%) are considered family‐owned and are small and medium‐sized operations, accounting for around 42% of the overall production value (Whitt 2020). These small and medium‐sized farms (SMFs) rely heavily on direct‐to‐consumer marketing channels, such as farmers markets, specialty food markets, and roadside stands, to sell their produce (Chen et al. 2021a). According to data that the U.S. Department of Agriculture published in 2021, around 8000 farmers markets are operating across the U.S. (Rakola 2021). In addition, the rise of online produce purchasing platforms has expanded market opportunities for SMFs. Farmers now have more access to the internet, which they use as a resource in their daily operations to seek and obtain information (Martinez and Park 2021), while consumers can go online to find information regarding produce that is fresh, local, and healthy. A project of virtual farmers markets in rural Ohio showed the potential of online platforms as a suitable option to reach more consumers and give them a different purchasing experience (Raison and Jones 2020). These trends suggest that produce from SMFs is increasingly accessible to consumers through various channels.

However, SMFs face challenges related to regulation compliance and meeting consumer expectations. Previous studies have reported that small‐scale farmers often struggle to comply with food safety principles due to the cost of compliance, limited time, and lack of resources (Chen et al. 2021a; Chen et al. 2021b). Thus, the U.S. Food and Drug Administration (FDA) exempts small produce farms, those with an average annual produce sales value of $25,000 or less over the past 3 years, from complying with the provisions of the Food Safety Modernization Act (FSMA) Produce Safety rule (U.S. Food and Drug Administration 2024a). Yet, most consumers believe that all farms, regardless of their size, should be subject to regulation (Ellison et al. 2016). Wu et al. (2021) indicate that consumers often rely on product assurance information, such as certifications, food attribute claims, and traceability information, to establish trust in both the stakeholders in the supply chain and the perceived safety and quality of the food products. This discrepancy between regulation requirements and consumer expectations may affect how consumers perceive the safety of produce from SMFs.

Consumers’ expectations and perceptions can be influenced by various product attributes. In today's world of agriculture and dietary habits, the origin of produce is at the forefront of consumer interest. This phenomenon goes beyond mere preference, tapping into the deep‐seated expectations and perceptions of risk that consumers associate with the safety and quality of their food. With a growing consciousness about how their food choices are interrelated with health and the environment, consumers are paying closer attention to where and how their produce is grown and processed. This shift in consumer behavior underscores a critical focus on the provenance and handling of farm produce, especially from smaller agricultural operations. Consumers who purchase produce from farmers markets directly interact with the growers, whose health‐oriented attitudes and behaviors drive the purchasing decisions of many consumers in this marketing channel (Stewart and Dong 2018).

In making purchase decisions of fresh produce, consumers consider both sensory attributes, such as freshness, appearance, and taste (Moser et al. 2011), which are associated with product quality, and non‐sensory attributes, such as local production (Fan et al. 2019), which are often linked to produce grown and sold by SMFs. For example, Batziakas et al. (2019) found that consumers preferred the flavor and texture of locally grown spinach produced in a high tunnel compared to commercially grown spinach. Moreover, the relative importance of these attributes that consumers consider when purchasing produce could vary across different demographic groups, further influencing consumer preferences (Gunden and Thomas 2012).

Even though sensory quality remains a key factor in consumer decisions, food safety has received increasing attention from consumers (Wu et al. 2021). Consumers today are more attuned to produce safety, due to news reports about multiple outbreaks and recalls linked to fresh produce in recent years (Centers for Disease Control and Prevention 2022; U.S. Food and Drug Administration 2022). Hence, they are paying closer attention to the microbial safety of their produce and expressing concern about pathogens such as *Escherichia coli* (Neill and Holcomb 2019). Gedikoglu and Gedikoglu (2021) reported that consumers value food safety information related to produce. However, data gaps persist regarding the influence of produce safety on consumers’ purchasing decisions when buying from SMFs.

Understanding these consumer expectations and perceptions is essential for addressing safety concerns and fostering trust in the agricultural products that SMFs offer to the market. The objective of this study is to assess consumers’ perceptions and expectations regarding produce from SMFs. Three types of widely consumed produce were included in this study: bell peppers, spinach, and kale (Lawrence 2022). These commodities have all been associated with several foodborne illness outbreaks and recalls in recent years (Food Safety News 2024; NBC News 2024; U.S. Food and Drug Administration 2018, 2021, 2022). Thus, they were used as a case study in the present study.

## 2 Materials and Methods

### 2.1 Pilot Study

The research protocol (IRB 2020‐1493) for this study was approved by the Institutional Review Board (IRB) at Purdue University (West Lafayette, IN, USA). The researchers developed most of the questions while adapting others from previous food safety studies. Ten consumers were interviewed to determine face validity. The criteria for eligibility were: (1) primary meal preparers and (2) those who had purchased any fresh peppers, spinach, or kale in the past month.

Participants’ feedback was recorded and analyzed to make further edits to the survey. A few questions were added based on participants’ suggestions, while others were reworded or deleted due to wording confusion. Concepts categorized as “too technical” were rephrased to enhance readability and thereby increase participants’ understanding. As a result of the revisions of the survey, a soft launch was conducted among 50 consumers to validate the flow of the survey. The soft launch of the survey revealed that one question was not displayed, and further modifications were made to rectify that omission.

### 2.2 Participant Recruitment

Participants were recruited between October and November 2021 by Qualtrics XM (Provo, UT), a third‐party online survey company. The researchers contracted Qualtrics to recruit participants from their online consumer panel. Qualtrics distributed survey invitations to their panel (U.S. citizens and/or residents) across the United States. Although the participants agreed to be contacted by Qualtrics, the researchers from this study added an agreement statement to ensure that all participants were willing to participate in the study. Those who selected “I do not agree to participate” were directed to the end of the survey.

Participants who completed the survey received an incentive provided by Qualtrics. The type and amount of the incentives were managed by Qualtrics and were not disclosed to the researchers.

### 2.3 Survey Design

The survey contained 89 questions, but some of the questions were visible only if the participant selected specific answers. The median survey completion time, based on the soft launch conducted (*n* = 50), was 9.75 min. Leiner (2019) suggests questionnaire completion time as a reliable indicator for detecting careless responses in online surveys. To ensure data quality, participants who completed the survey in less than 4.8 min (half of the completion time from the soft launch) were excluded from the present study. This decision aligns with Leiner's (2019) recommendation to use relative completion speed (a pragmatic cut‐off of completing the survey at twice the typical speed) as a criterion for identifying suspicious data. The survey encompassed three types of questions: (1) multiple‐choice, including the option to allow one or multiple answers; (2) a five‐point Likert scale; and (3) a ten‐point line scale. Some specific answers required text entry to clarify choices; however, that format was limited to decrease the participants’ fatigue. Appendix A lists all questions.

### 2.4 Participant Screening and Demographic Questions

Participant inclusion criteria for the survey were: (1) primary grocery shopper of the household; and (2) those who purchased any bell peppers, spinach, or kale in the past month. The survey asked two screening questions: “Are you the primary grocery shopper for yourself and/or your household?” and “Have you purchased any of the following fresh produce in the past month: bell peppers, spinach, or kale (not canned, frozen, or in a ready‐to‐eat meal)?” Only those who answered “Yes” to both questions were allowed to continue with the survey.

To ensure that the sample (*n* = 916) was representative of the U.S. population, the survey sampling was quota‐controlled for the state of residence, gender, race and ethnicity, education level, household income, and area. The answer choices for the demographic question concerning the area where participants live were either “urban,” “suburban,” or “rural.” The participants were asked four demographic questions that were not included in the quotas: “Do you live with children under the age of 18, children under the age of 5, or adults aged 65 or older?” as well as “Do you consider yourself vegetarian or vegan?” For the last question, definitions of both concepts were provided.

### 2.5 Purchasing and Handling Practices

To identify participants’ practices for purchasing and handling bell peppers, kale, and/or spinach, the survey asked 13 questions. The questions were related to where participants usually purchase their produce, how they handle their produce at the market and at home, as well as the preparation or cooking methods for consumption of their produce. This section helped to generate consumers’ profiles.

### 2.6 Produce Food Safety Knowledge and Expectations

Four questions sought to identify participants’ produce safety knowledge, with possible responses of “true,” “false,” or “I don't know.” These questions evaluated whether consumers knew how pathogenic bacteria can be transferred from the farm to the produce they purchase. A food safety knowledge score was calculated for each participant based on their total number of correct responses to the knowledge questions, with the total score ranging from 0 to 4. Additionally, one question assessed whether they considered food safety a “minimum quality standard,” and if SMFs should comply with the same food safety rules as larger farms.

### 2.7 Food Safety Risk Attitudes and Perceptions

To understand participants’ food safety risk attitudes, which refer to their willingness to take risks in decision‐making, five questions were asked concerning their health, and financial decisions as well as eating habits. The questions were evaluated using a ten‐point line scale with anchors at 0 and 10, representing “not at all willing to take risks” to “very willing to take risks.” This scale allows for the capture of a broader and more continuous range of responses.

The survey also assessed participants’ food safety risk perceptions, which refer to their perceptions of the likelihood of potential food safety hazards. The survey posed seven questions regarding the extent to which participants considered hazards, production locations, and labels on produce to be important factors when assessing the food safety risks associated with produce purchases. Participants were asked to respond using a five‐point Likert scale from “not at all important” to “extremely important.” A further seven questions examined participants’ perceptions of the degree to which produce safety is affected by various supply chain entities and procedures, including government agencies, farmers, produce transportation methods, produce packing facilities, retailers, chefs, and meal preparers, as well as consumers themselves. These questions were evaluated using a five‐point Likert scale from “not at all influential” to “extremely influential.” Finally, participants were asked to indicate which actors or procedures in the supply chain might have caused their exposure to pathogenic contamination in the event of a hypothetical outbreak.

### 2.8 Perceptions of SMFs

To understand participants’ perceptions of the produce sold by SMFs, six questions were asked about the degree to which various produce grower characteristics influence their decisions when making purchases. These questions were evaluated using a five‐point Likert scale from “not at all important” to “extremely important.” Participants were asked one question about the difference between produce from SMFs compared to large farms. These questions were divided into procurement, availability, and food safety, and answers were evaluated with a five‐point Likert scale from “strongly agree” to “strongly disagree.” Finally, consumers were randomly shown two headlines regarding 50 people becoming sick from spinach grown on a small or large farm. The level of concern from these headlines was evaluated with a 5‐point Likert scale from “very concerned” to “not concerned at all.”

### 2.9 Statistical Analysis

Descriptive analysis was conducted to include frequencies and percentages. Logistic regression was conducted to analyze the question about food safety as a minimum quality standard and the level of influence of each actor in the supply chain. The sociodemographic characteristics were the independent variables used for the logistic regression. Both descriptive analysis and logistic regression were analyzed using IBM SPSS Statistics version 27.0 (IBM, Armonk, NY, USA) with a confidence interval of 95% (*P* ≤ 0.05).

To further determine the interrelationships of various constructs in the present study, structural equation modeling (SEM) was conducted, using the Lavaan package (version 0.6‐19) in R (version 4.5.1). The constructs examined included demographic characteristics (age, gender, household income, education level, living area, living with young children, living with older adults, and eating habit as vegetarian or vegan), food safety knowledge score, expectation of food safety as a minimal quality standard, produce handling practices, perceptions of produce from SMFs, and perceptions of food safety standards for SMFs. Two latent constructs were produced: handling practices (measured by self‐reported produce handling practices relating to separate, washing, storing, and cooling), and perceptions of produce from SMFs (measured by perceived freshness, quality, and safety of produce from SMFs). As the majority of the data used in the model were categorical or ordinal, the weighted least squares mean and variance adjusted (WLSMV) was used as an estimator. Statistical significance was determined at a significance level of 0.05 (*p* < 0.05).

## 3 Results

### 3.1 Participants’ Profile

The sample consisted of 916 participants who identified themselves as the primary grocery shoppers in their households and indicated that they had purchased fresh bell peppers, spinach, or kale in the past month (see Table 1 for sociodemographic details). Participants were mostly female, white, non‐Hispanics, had at least some college education, and lived in suburban areas. Some participants lived with high‐risk population groups: children under 5 years, or adults 65 years and above.

**TABLE 1:** Sociodemographic characteristics of survey participants (*n *= 916).

Characteristics	Response *N* (%)
*Gender*	
Male	419 (46)
Female	497 (54)
*Age group*	
18–24	130 (14)
25–34	180 (20)
35–44	170 (19)
45–54	151 (16)
55–64	121 (13)
65 and above	164 (18)
*Race/Ethnicity* ^a^	
White non‐Hispanic	589 (64)
Hispanic or Latino	224 (24)
Asian or Pacific Islander	28 (3)
Native American	13 (1)
African American	128 (14)
Other	9 (1)
*Education level*	
Less than high school diploma/GED	47 (5)
High school diploma/GED	280 (30)
Some college (no degree)	210 (23)
Associate's degree	80 (9)
Bachelor's degree	189 (21)
Graduate degree	110 (12)
*Household's income*	
Less than $25,000	180 (20)
$25,000–$49,999	219 (24)
$50,000–$74,999	190 (21)
$75,000–$99,999	96 (11)
$100,000–$149,999	111 (12)
$150,000–$199,999	60 (6)
$200,000 and above	60 (6)
*Community*	
Urban region	269 (29)
Suburban region	450 (49)
Rural region	197 (22)
*Children*	
Under 18	360 (39)
5 years or younger	184 (20)
*Adults*	
65 years or older	236 (26)
*Vegetarian or Vegan*	203 (22)

^a^ Race/ethnicity questions allowed to choose multiple answers.

Table 2 shows participants’ produce purchasing and handling practices for bell peppers (*n* = 792), spinach (*n* = 593), and kale (*n* = 327). Participants purchased their produce from any of three places: local supermarkets, farmers markets, or specialized markets. One‐sixth of the participants used online platforms and apps to purchase produce.

**TABLE 2:** Participants’ produce purchasing and handling practices.

Handling practices	Response *n* (%)
*Purchasing practices* (*n* = 916)	
Local supermarkets	741 (81)
Farmers markets	416 (45)
Specialized markets (e.g., Fresh Thyme, Market District, Whole Foods Market)	264 (29)
Roadside stands	197 (22)
Online grocery delivery apps (e.g., Instacart, local supermarket app)	165 (18)
Online websites (e.g., Imperfect Foods, Misfit Market, Farmbox, Amazon Fresh)	144 (16)
Directly from the farms	120 (13)
CSA (Community supported agriculture)	73 (8)
None of the options	10 (1)
*Handling practices*	
Bell peppers preparation (*n* = 792)	
Boiling	187 (24)
Steaming	275 (35)
Blanching	100 (13)
Roasting	360 (45)
Stir‐frying	516 (65)
Grilling	394 (50)
I eat them raw	298 (38)
Other	11 (1)
Kale preparation (*n* = 327)	
Boiling	124 (38)
Steaming	150 (46)
Blanching	85 (26)
Roasting	104 (32)
Stir‐frying	130 (40)
Grilling	76 (8)
I eat it raw	114 (35)
Other	7 (2)
Spinach preparation (*n* = 593)	
Boiling	217 (37)
Steaming	288 (49)
Blanching	103 (17)
Roasting	121 (20)
Stir‐frying	226 (38)
Grilling	97 (16)
I eat it raw	306 (52)
Other	12 (2)
I make sure the bell peppers that I purchase are firm and not bruised (*n* = 792)	
Never	13 (2)
Sometimes	151 (19)
Always	628 (79)
I make sure the spinach or kale that I purchase is not wilted or turning a different color (*n* = 657)	
Never	10 (2)
Sometimes	113 (17)
Always	534 (81)
I separate the vegetables from raw meat, chicken, or fish at the market, using individual bags (*n* = 916)	
Never	33 (4)
Sometimes	159 (17)
Always	724 (79)
I typically pack a cooler, insulated bag, or ice packs when planning to purchase produce (*n* = 916)	
Never	382 (42)
Sometimes	299 (32)
Always	235 (26)
I wash the produce that I purchase if it is not in a container that says it was pre‐washed (*n* = 916)	
Never	31 (3)
Sometimes	201 (22)
Always	684 (75)
I store the following produce: bell peppers, kale, and/or spinach inside the refrigerator (*n* = 916)	
Never	24 (3)
Sometimes	154 (16)
Always	738 (81)
I store the vegetables in a part of the refrigerator that is separated from raw meat, chicken, or fish (*n* = 856)^a^	
Never	8 (1)
Sometimes	118 (14)
Always	730 (85)

^a^ This question was displayed only according to the logic flow of “I store the following produce: bell peppers, kale, and/or spinach inside the refrigerator.” Additionally, due to initial problems in the flow of the survey, this question was not displayed to some participants.

More than a third said they eat raw bell peppers and raw kale, and more than half eat raw spinach. Most bell pepper buyers always make sure that the bell peppers they are buying are firm and not bruised, and the leafy greens buyers ensure that their kale and/or spinach is not wilted or turning a different color.

Most participants make sure to separate their produce from raw meat, chicken, or fish, using individual bags while shopping. But only around one‐fourth reported always packing a cooler, insulated bag, or ice packs when planning to purchase produce. At home, most of the participants always wash their produce items if they are not pre‐washed; they store bell peppers, kale, and/or spinach inside the refrigerator; and ensure that produce items are in a part of the refrigerator that is separated from raw meat, chicken, or fish.

### 3.2 General On‐Farm Food Safety Knowledge of Bell Peppers, Kale, and Spinach Consumers

**FIGURE 1:**
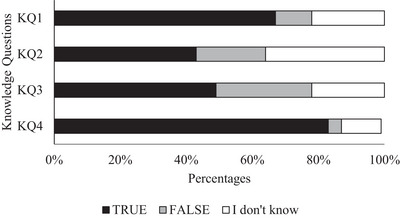
Participants’ knowledge of pathogen transfer from farm to the produce they purchase. KQ1: At the farm, soil and water can be the source of disease‐causing microorganisms that can contaminate produce. KQ2: Farmers can use raw manure as a soil amendment immediately before the harvest of leafy greens, like spinach. KQ3: Damaged or bruised produce does not have a higher risk of causing foodborne illness if the produce is properly washed before consumption. KQ4: On‐farm food safety practices can help reduce the risk of produce from carrying harmful bacteria.

Sixty‐seven percent of participants knew that soil and water at farms could be vehicles for pathogenic bacteria and could potentially contaminate their produce (Figure 1). Also, 83% knew that on‐farm food safety practices could reduce the risk of contamination by pathogenic bacteria. However, two incorrect answers included in this survey were surprisingly selected as “true” by some participants. Approximately 43% of the participants think that using raw manure as a soil amendment immediately before the harvest of leafy greens is a correct practice. Moreover, almost half of the participants think that bruised produce does not have a higher risk of causing foodborne illness if they properly wash it.

### 3.3 Food Safety as a Minimum Quality Standard and Attributes Considered When Assessing the Food Safety Risk of Produce

Eighty‐five percent of participants considered food safety as the minimum quality standard of the produce they purchased. The main focus of this study was to determine the association between sociodemographic characteristics and the consumer's belief that produce safety is a minimum quality standard when purchasing produce. Table 3 shows that certain subgroups within the sociodemographic groups considered food safety as a minimum quality standard.

**TABLE 3:** Logistic regression regarding produce safety as a minimum quality standard among sociodemographic characteristics.

Sociodemographic	Likelihood ratio test (*P* value)	*P* value	OR	95% CI
*Gender*	0.21			
Female		0.21	0.79	(0.54; 1.14)
Male (baseline)		—	—	—
*Age range*	0.13			
18–24		0.26	0.68	(0.35; 1.33)
25–34		0.29	0.71	(0.38; 1.33)
35–44		0.91	1.04	(0.53; 2.05)
45–54		0.54	0.81	(0.42; 1.58)
55–64		0.017*	0.46	(0.24; 0.87)
65 and above (baseline)		—	—	—
*Education level*	0.01*			
Less than high school diploma/GED		0.42	1.43	(0.60; 3.41)
High school diploma/GED		0.48	1.18	(0.75, 1.86)
Associate's degree		0.05*	2.25	(1.01; 5.03)
Bachelor's degree		0.07	2.24	(1.25; 4.00)
Graduate degree		0.01*	2.50	(1.20; 5.21)
Some college (no degree) (baseline)		—	—	—
*Household income*	0.02*			
$25,000–$49,999		0.57	1.17	(0.68; 2.01)
$50,000–$74,999		0.86	0.95	(0.55; 1.64)
$75,000–$99,999		0.52	0.81	(0.43; 1.53)
$100,000–$149,999		0.47	1.28	(0.66; 2.50)
$150,000–$199,999		0.02*	5.80	(1.34; 25.05)
$200,000 and above		0.06	2.80	(0.94; 8.31)
Less than $25,000 (baseline)		—	—	—
*Area*	0.001*			
Rural		<0.001*	0.39	(0.23; 0.65)
Suburban		0.10	0.67	(0.42; 1.09)
Urban (baseline)				
*Living with children*	0.05			
No children aged 5 or younger		0.06	0.61	(0.37; 1.02)
Children aged 5 or younger (baseline)		—	—	—
*Living with elderly*	0.21			
No adults 65 years or older		0.22	0.76	(0.49; 1.18)
Adults 65 years or above (baseline)		—	—	—
*Eating habits*	<0.001*			
No vegetarians or vegans		<0.001*	0.36	(0.20; 0.64)
Vegetarians or vegans (baseline)		—	—	—

* *P* ≤ 0.05.

The logit model considered the following sociodemographic characteristics: education level, household income, living area, and eating habits. Based on the education level, people with university diplomas, including associate degrees, bachelor's degrees, and graduate degrees, were more likely to consider food safety as a minimum quality standard than those with some college education but no degree, as shown in Table 3: 2.25 (confidence interval 1.01; 5.03), 2.24 (confidence interval 1.25; 4.00), and 2.50 (confidence interval 1.20; 5.21), respectively.

Participants with a household income between US$150,000 and US$199,999 were 5.80 times more likely (confidence interval 1.34; 25.05) to consider food safety as a minimum quality standard than those with a household income less than US$25,000 (Table 3). In contrast, participants living in rural areas considered food safety as a minimum quality standard less frequently (odds ratio 0.39; confidence interval 0.23; 0.65) (Table 3). In addition, nonvegetarian or nonvegan participants were less likely than vegetarian or vegan respondents to consider food safety as a minimum quality standard (odds ratio 0.36; confidence interval 0.20; 0.64) (Table 3).

**FIGURE 2:**
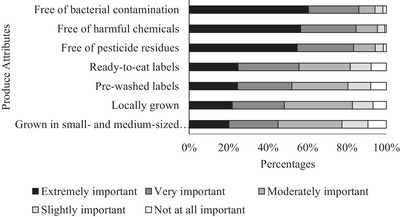
Produce attributes considered when assessing the food safety risk of produce purchased. (Each characteristic was measured on a five‐point Likert‐type scale).

The majority of consumers believe that the lack of bacterial contamination, harmful chemicals, and pesticide residues is extremely important when assessing the food safety risk of produce, by 61%, 56%, and 55%, respectively. Nevertheless, the labels indicating that produce is local, has been pre‐washed, or is ready to eat are perceived as important, but not as important as the absence of pesticide residues, harmful chemicals, or bacterial contamination (Figure 2).

### 3.4 Responsibility for Produce Safety

**FIGURE 3:**
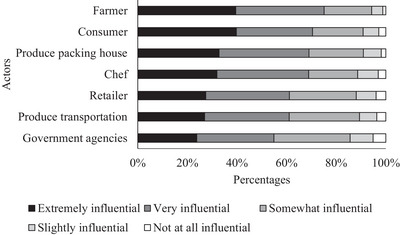
Level of influence of the supply chain actors in produce safety.

**FIGURE 4:**
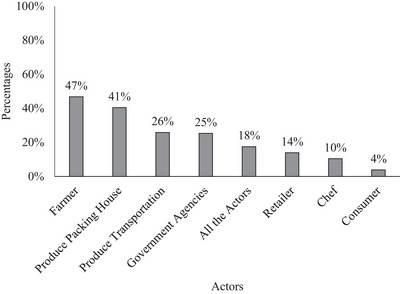
Responsible actors in the “Multistate Outbreak of Shiga Toxin‐Producing *Escherichia coli* 0157:H7 Infections Linked to Organic Spinach and Spring Mix Blend.” (One consumer could choose multiple actors.)

As shown in Figure 3, consumers rated the impact of different groups along the supply chain on produce safety. Most consumers believe that all actors involved in the produce safety chain (government agencies, farmers, produce transport personnel, packing houses, retailers, chefs, and consumers) have some level of influence. However, 40% of consumers believe that farmers and consumers themselves are “extremely influential” with regard to the safety of produce. Appendix B shows how the sociodemographic characteristics could predict the rate attributed to each of the actors for their influence on produce safety. When considering the influence of farmers (Table 4), the sociodemographic age of respondents was significant to the logit model. Those aged 18 to 24 and 25 to 34 considered farmers to be less influential in produce safety than did those aged 65 years and above. Additionally, for them as consumers, the sociodemographic age and living area were significant. Those aged 25 to 34 considered farmers to be less influential on produce safety than did those aged 65 and older. Consumers living in suburban areas considered farmers to have less influence on produce safety than did those living in urban areas. Also, when consumers were presented with the headline “Multistate Outbreak of Shiga Toxin,” 47% blamed the farmer for the outbreak (Figure 4).

**TABLE 4:** Logistic regression regarding the level of influence of “Farmer” in produce safety.

Sociodemographic	Likelihood ratio test (*P* value)	*P* value	OR	95% CI
*Gender*	0.841			
Female		0.841	0.974	(0.751; 1.263)
Male (baseline)		—	—	—
*Age range*	0.011^*^			
18–24		0.034^*^	0.585	(0.356; 0.959)
25–34		0.003^*^	0.509	(0.325; 0.798)
35–44		0.222	0.754	(0.479; 1.186)
45–54		0.094	0.691	(0.448; 1.064)
55–64		0.644	1.113	(0.707; 1.752)
65 and above (baseline)		—	—	—
*Education level*	0.191			
Less than high school diploma/GED		0.296	0.676	(0.324; 1.410)
High school diploma/GED		0.091	0.653	(0.399, 1.071)
Some college (no degree)		0.462	0.828	(0.501; 1.368)
Associate's degree		0.024	0.501	(0.276; 0.912)
Bachelor's degree		0.122	0.702	(0.448; 1.100)
Graduate degree (baseline)		—	—	—
*Household income*	0.587			
Less than $25,000		0.360	1.335	(0.719; 2.478)
$25,000–$49,999		0.657	1.144	(0.631; 2.073)
$50,000–$74,999		0.191	1.483	(0.821; 2.679)
$75,000–$99,999		0.179	1.554	(0.817; 2.954)
$100,000–$149,999		0.199	1.477	(0.815; 2.676)
$150,000–$199,999		0.201	1.542	(0.794; 2.992)
$200,000 and above (baseline)		—	—	—
*Area*	0.055			
Rural		0.375	0.840	(0.572; 1.234)
Suburban		0.019^*^	0.690	(0.506; 0.941)
Urban (baseline)		—	—	—
*Living with children*	0.076			
No children aged 5 or younger		0.076	0.741	(0.531; 1.032)
Children aged 5 or younger (baseline)		—	—	—
*Living with elderly*	0.452			
No adults aged 65 or above		0.452	0.892	(0.661; 1.202)
Adults aged 65 or above (baseline)		—	—	—
*Eating habits*	0.667			
No vegetarians or vegans		0.667	1.072	(0.782; 1.468)
Vegetarians or vegans (baseline)		—	—	—

*Note*: To interpret the results, the model was performed with an ascending parameter for the dependent variable.

* *P* ≤ 0.05.

### 3.5 Participants’ Perceptions Regarding SMFs

Consumers were asked to respond to a series of questions about their perceptions of SMFs (Table 5). Most consumers conceived of SMFs as family‐owned and yielding US$30,000 or less in annual average value of produce sold. In addition, most of respondents believe that SMFs should follow the same food safety rules required for larger farms. Moreover, after consumers were presented with a general definition of the Produce Safety Rule (PSR) and the exemption of small farms, 63% said they opposed exempting small farms from the regulation. Among those who disagree with the exemption of small farms, 74% reported that all farms should be regulated, and 67% indicated that all commercially sold produce must be safe for consumption.

**TABLE 5:** Consumers’ general perception of small and medium‐sized farms.

Question	Response *n* (%)
*Definition of small and medium‐sized farms*	
Farms that have an average annual value of produce sold of $30,000 or less	371 (41)
Farms that have an average annual value of produce sold of $575,000 or less	314 (34)
Farms that have an average annual value of produce sold between $575,000 and $1 M (one million)	111 (12)
Farms that are family‐owned	375 (41)
I have never thought about it	208 (23)
None of the above, but other (please specify)	1 (<1)
*Should small or medium‐sized farms have to follow the same food safety rules as larger farms?*	
Yes	781 (85)
No	43 (5)
Not certain	92 (10)
*Do you agree that small farms should be exempt from food safety regulations?* ^a^	
Yes	310 (33)
No	574 (63)
Not certain	32 (4)
*Why do you disagree that small farms should be exempt from food safety regulations?* (*n* = 574)	
All farms should be regulated in the same way	423 (74)
The produce has to be safe for consumption	382 (67)
The food safety regulations are easy to follow	161 (28)
*Why do you agree that small farms should be exempt from food safety regulation?* (*n* = 310)	
They are operated by families	170 (55)
They benefit the community	187 (60)
Their production is small	178 (57)
We have to support local produce growers	116 (37)

^a^ This question was asked to consumers after they read a paragraph about the Produce Safety Rule and some of the exemptions of small farms.

**FIGURE 5:**
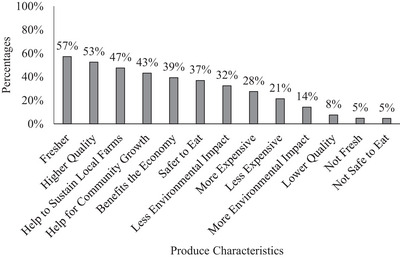
Characteristics of produce purchased from small and medium‐size farms that differ from large farms. (One consumer could choose multiple actors).

Consumers identified some characteristics that they believe differentiate the produce sold by SMFs from produce sold by large farms (Figure 5). Consumers consider the produce sold by SMFs as fresher (57%) and of higher quality (53%) than produce grown on large farms, and 47% think their purchases help to sustain local farms. However, only 37% believe that produce sold by SMFs is safer to eat.

### 3.6 Interrelationships Among Various Constructs

**FIGURE 6:**
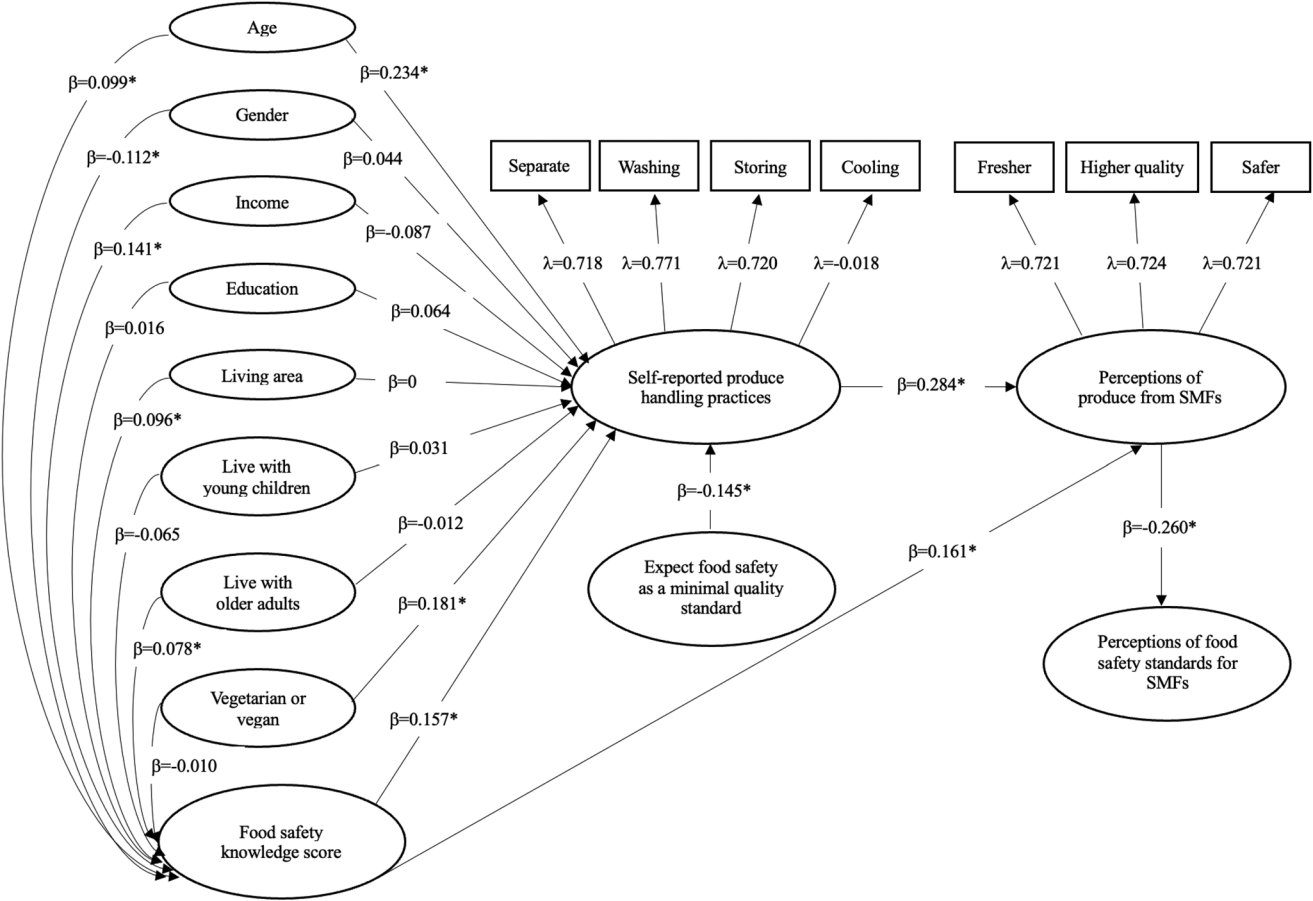
Path diagram illustrating the relationships among demographic characteristics, self‐reported produce handling practices, food safety expectation, food safety knowledge score, perceptions of produce from small‐ and medium‐sized farms (SMFs), and perceptions of food safety standards for SMFs. Symbol λ represents factor loadings, and symbol *β* represents standardized path coefficients. Asterisks (*) indicate paths that are statistically significant at a significance level of 0.05 (*p* < 0.05).

Figure 6 presents the path diagram illustrating the relationships among demographic characteristics, self‐reported produce handling practices, food safety expectations, food safety knowledge score, perceptions of produce from SMFs, and perceptions of food safety standards for SMFs. The model demonstrated acceptable fit to the data, with a root mean square error of approximation (RMSEA) of 0.052 and a standardized root mean square residual (SRMS) of 0.08, consistent with the fit indices suggested by Hooper et al. (2008). The model showed that age (*β* = 0.234, *p* < 0.001), being vegetarian or vegan (*β* = 0.234, *p* < 0.001), and food safety knowledge score (*β* = 0.157, *p* < 0.001) were significantly positively associated with safe produce handling practices. In contrast, consumers’ expectation that food safety should be a minimum quality standard of the produce they purchased (*β* = −0.145, *p* = 0.001) was significantly negatively associated with safe produce handling practices, suggesting that consumers who perceived food safety as a minimal quality standard were less likely to report adopting safe produce handling practices.

While not directly associated with safe produce handling practices, some demographic characteristics, including gender (*β* = −0.112, *p* = 0.002, male = 0, female = 1), income level (*β* = 0.141, *p* = 0.002), living area (*β* = 0.096, *p* = 0.013, urban = 1, suburban = 2, rural = 3), and living with older adults (*β* = 0.078, *p* = 0.038), were significantly associated with food safety knowledge score, which had a significant positive impact on produce handling practices.

Both food safety knowledge score (*β* = 0.161, *p* < 0.001) and produce handling practices (*β* = 0.284, *p* < 0.001) were significantly positively associated with perceptions that produce from SMFs was fresher, of higher quality, and safer, which, in turn, was significantly negatively associated with the perceptions of food safety standards for SMFs. This result suggested that those who perceived produce from SMFs as fresher, of higher quality, and safer were less likely to perceive that SMFs should follow the same food safety rules required for larger farms.

## 4 Discussion

### 4.1 Food Safety as a Minimum Requirement

As demand for quality and safe produce increases, consumers are paying more attention to attributes of the produce, such as whether it has been grown conventionally or organically (Bucholtz 2020). About 85% of consumers who participated in this study expect that the produce they purchase is safe for consumption—free of bacteria, harmful chemicals, and residues of pesticides—and they consider food safety to be a minimum quality standard.

According to a recent study, consumers are concerned about the possible presence of *E. coli* in the produce they purchase (Neill and Holcomb 2019). Additionally, Ortega et al. (2011) indicated that consumers are increasingly concerned about the safety of food products. According to their study, Chinese consumers have a high level of food safety risk perception, which could be attributable to certain food scandals involving Chinese food products. In the United States, between 2010 and 2017, a total of 83 multistate outbreaks of pathogenic *E. coli*, *Salmonella enterica*, and *Listeria monocytogenes* have been linked to fresh produce such as melons, sprouts, leafy greens, and peppers sold in the United States (Carstens et al. 2019).

A number of fresh produce recalls have also been reported recently (Paramithiotis et al. 2017). Moreover, news broadcasts and social media coverage have increasingly exposed consumers to information regarding food outbreaks and recalls (Nasheri et al. 2019). Consumers who were presented with a hypothetical headline, “Multistate Outbreak of Shiga Toxin‐Blend,” attributed a level of responsibility (“very influential” and “extremely influential”) to all the actors involved. However, nearly half of the study participants (47%) believe that the main responsibility rests with farmers. This concurs with Khouryieh et al. (2019), who found that growers were primarily responsible for perishable products sold at farmers markets to consumers. In addition, consumers perceived themselves as responsible for the safety of these perishable products. Henke et al. (2021) obtained similar results, but their study was conducted among German meat (poultry) consumers. That study found that when German consumers assess the safety of meat, they consider every party involved in the supply chain to have a certain degree of responsibility. Most consumers (70%) believed that operators of poultry fattening farms had a “high” or “very high” level of responsibility. Thus, growers of every size are found to have a high level of responsibility for any food safety outbreak.

### 4.2 Consumer Practices and Knowledge Gaps in Produce Safety

Consumers play a crucial role in ensuring produce safety, yet many fail to adopt proper handling practices or fully understand the risks associated with improper storage, consumption of raw produce, and the use of contaminated inputs. Consumers have high expectations for the safety of produce; however, not all consumers are doing their part to ensure produce safety. Some consumers engage in improper handling practices and do not possess a detailed understanding of on‐farm growing and food safety procedures (Yu et al. 2022). The SEM results in the present study showed that consumers who considered food safety as a minimal quality standard were less likely to report safe produce handling practices. This could be due to consumers’ trust in the stakeholders in the food system in ensuring the safety of their produce; thus, they pay less attention to their own handling practices. A previous study also reported that food handlers relied on their suppliers in ensuring product safety, and therefore were not concerned about food safety themselves (Chen et al. 2024).

When foods are contaminated with pathogens and transported uncooled over a long distance, the risk of foodborne illness increases. Only 26% of our respondents always packed a cooler, insulated bag, or ice packs when they purchased produce. This concurs with another study in which only 16% of respondents surveyed packed a cooler, insulated bag, ice chest, or ice packs when they purchased produce (Khouryieh et al. 2019). These findings were correlated with the amount of time between consumers’ purchase of perishables and their refrigeration. Consumers who did not bring cooling bags or chests with them when purchasing produce refrigerated their perishables within an hour of purchasing them. However, those who took cool storage items with them on their shopping trips were more likely to delay refrigeration of their perishables by 2 h or longer after purchasing them. Even though most consumers refrigerate produce items within an hour of purchasing them, the risk of contracting foodborne illness is higher if they do not pack a cooler or any other temperature‐control item and delay refrigeration of their produce for 2 h or more after purchase.

Additionally, some consumers reported eating fresh raw bell peppers (38%) and raw kale (35%), while more than half of the consumers reported eating raw spinach (52%). This is another practice that may increase the risk of contracting a foodborne illness. Despite consumers washing their produce that lacked a prewashed label, the act of eating raw produce increases the risk of microbial contamination. For example, food handlers who have hepatitis A can transmit the virus to produce, which will act as a vehicle for the foodborne pathogen (Fung et al. 2018).

Leafy greens sometimes have a high microbial load that can lead to foodborne illness (Bimal Sheth et al. 2025). Moreover, the CDC also reported that a total of 51 foodborne outbreaks between 2014 and 2018 occurred due to eating contaminated leafy greens. The consumption of raw leafy greens has been responsible for a high percentage of foodborne illness, for which norovirus is a pathogen of concern (Painter et al. 2013). Well‐established companies have been responsible for some of these outbreaks (Centers for Disease Control and Prevention 2016). However, the produce sold at farmers markets by DTC (direct‐to‐consumer) farms is a potential point of fresh produce outbreaks (Bellemare and Nguyen 2018), some of which have been documented. Outbreaks that have been associated with farmers markets are linked to strawberries and peas (Food Safety News 2017; Gardner et al. 2011; Goetz 2011). In addition, studies have evaluated the microbial quality of produce from these selling points. Scheinberg et al. (2017) took numerous samples from produce and meat at a farmers market in Pennsylvania and found the prevalence of *E. coli* and *Listeria* spp., indicator microorganisms, in certain samples collected.

Also, Pan et al. (2015) found a prevalence of fecal coliform, *E. coli*, and *Salmonella* in certain samples of six agricultural commodities. The U.S. Food and Drug Administration has given recommendations to consumers on how to handle their produce (U.S. Food and Drug Administration 2024b). For leafy greens that will be eaten raw, the guidelines suggest (1) washing hands before and after preparing leafy greens, (2) removing any wilted or bruised leaves, (3) rinsing leaves under running water, and (4) drying using a clean towel or paper towel.

In addition, some consumers lack knowledge related to the safety of their produce. Almost half do not believe that bruised produce presents a higher risk of contracting a foodborne illness if it is properly washed. In fact, porous tissue (stomata and lenticel), bruises, cuts, or any crack in the produce surface can enhance foodborne pathogens’ attachment and promote their colonization (Alegbeleye et al. 2018). Bruises or tissue damage give a route to microorganisms to internalize into produce (Kaczmarek et al. 2019). Moreover, 43% of consumers who responded believe that manure can be applied to leafy greens before harvesting. This is not recommended because it may lead to an increased risk of microbial contamination of fresh produce. Several pathogenic microorganisms are naturally present in raw manure, including *E. coli*, *Salmonella* spp., *Campylobacter* spp., and *Listeria monocytogenes* (Manyi‐Loh et al. 2016). Raw manure must be incorporated 120 days or more before harvesting if the edible parts of the product are in contact with the soil (Code of Federal Regulations 2022). In addition, manure may be incorporated no more recently than 90 days before harvesting if the edible parts of the product are not in contact with the soil.

The SEM results showed a significant positive relationship between food safety knowledge score and safe produce handling practices. This finding aligns with Yu et al. (2022), who reported that consumers with higher food safety knowledge in the U.S. had significantly better food safety practices when handling fresh‐cut produce. The model in the present study also revealed that increasing age and being vegetarian or vegan were significantly positively associated with safe produce handling practices. A previous study has found that consumers tend to have higher food safety awareness as they get older (Parra et al. 2014), possibly due to their increased years of food handling experience and accumulated knowledge, which could result in better food handling practices. In addition, consumers who are vegetarians and vegans have been reported to be more health‐conscious, compared to those who do not follow the same diets, when making food‐related decisions (Bedford and Barr 2005); therefore, they might also be more cautious when it comes to food safety practices.

### 4.3 Consumers’ Perception of Produce Sold by SMFs

As a result of this study, most consumers believe that SMFs should adhere to the same food safety regulations as large farms. Moreover, when consumers were exposed to the Produce Safety Rule definition and small farm exemption policy, the majority disagreed with the exemption of these farms, claiming that their produce should be subject to the same regulations as large farms in order to attain safety for consumption. Ellison et al. (2016) reported similar findings. After consumers were exposed to FSMA information, their perception of the safety of produce declined for certain markets, such as fresh format and farmers markets. The study also found that consumers strongly agree that all farms should be regulated the same way.

In addition, around half of the consumers in our study reported that the produce sold by SMFs is fresher and of higher quality than that of larger farms, and that buying from small growers supports local farmers. Despite this, only about 37% considered that produce from small farms was safer to eat. Khouryieh et al. (2019) found that 97% of consumers regard freshness and product taste as “very important” or “extremely important.” Another study revealed that consumers consider locally grown produce to be fresher, of higher quality, safer, and more nutrient‐dense than conventional produce (Onozaka et al. 2010). Crandall et al. (2011) found that 51% of consumers support local farmers by shopping at farmers markets. The study also found that only a small percentage of consumers (6%) believed the produce from farmers markets was safe. An analysis of content on a popular social media platform identified six major hashtags (keywords) used when searching for farmers markets: organic, fresh, food, local, vegan, and healthy (Pilař et al. 2018). The common findings from all the literature regarding the produce sold by SMFs, or markets in which SMFs sold their produce, they all refer to attributes of “freshness,” “quality,” and “locally grown,” but “safer” was not a common attribute among different studies. This suggests that consumers value other attributes not related to produce safety when purchasing produce from these farms.

In addition, the SEM model showed that consumers with perceptions that produce from SMFs was fresher, of higher quality, and safer, were significantly less likely to perceive that SMFs should follow the same food safety rules required for larger farms. Consumers with positive perceptions of produce from SMFs might trust these farms more than large farms. A previous study conducted by Busch et al. (2022) also reported that most consumers preferred small animal farms over the large ones and perceived products from small farms as having higher quality. Therefore, consumers might perceive less of a need for formal regulatory requirements for these SMFs.

## 5 Conclusion

This study assessed consumers’ perceptions and expectations regarding fresh produce safety and revealed a panorama of what consumers think about produce from SMFs. At first glance, some of the consumers’ handling practices of their fresh produce could increase their risk of contracting a foodborne illness. The study confirmed that consumers have some knowledge gaps in how farmers apply certain on‐farm food safety practices. Consumers consider food safety a minimum quality standard, and hence, they expect it when purchasing their fresh produce. Moreover, they attribute the safety of their produce to the farmer when a situation like a foodborne outbreak happens. Participants in this study consider produce grown and sold by SMFs to be fresher, of higher quality, and more supportive of the sustainability of local farms than produce grown on large farms. However, the majority did not agree with their exemption from the Produce Safety Rule and would like all farms, regardless of size, to comply with the regulation. This finding highlights the discrepancy between consumer expectations and regulatory requirements, suggesting opportunities for consumer food safety education to improve their knowledge, address misperceptions, and promote safe produce handling practices.

The researchers intend to share these findings with produce safety specialists and policymakers to help farmers protect the health of their consumers by beginning or continuing to implement food safety practices that are economically feasible. The findings can also be shared directly with farmers to raise their awareness of consumer expectations regarding food safety and to encourage the adoption of safe food production practices. Farmers could use technology, such as quick response codes, which consumers can scan with electronic devices to access additional digital content about farming practices and food safety procedures, to enhance transparency with their consumers and increase their competitiveness in the market. Future research could further explore effective communication strategies that help farmers convey food safety information to consumers.

## Author Contributions

**Juan Carlos Archila‐Godínez**: methodology, formal analysis, writing – original draft. **Claudia Kotanko**: writing – review and editing. **Renee Wiatt**: funding acquisition, writing – review and editing. **Maria I. Marshall**: funding acquisition, writing – review and editing. **Yaohua Feng**: conceptualization, methodology, supervision, writing – review and editing.

## Conflicts of Interest

The authors declare no conflicts of interest.

## Supporting information




**Supplementary Materials**: jfds70527‐sup‐0001‐Appendix.docx

## References

[jfds70527-bib-0001] Alegbeleye, O. O. , I. Singleton , and A. S. Sant'Ana . 2018. “Sources and Contamination Routes of Microbial Pathogens to Fresh Produce During Field Cultivation: A Review.” Food Microbiology 73: 177–208. 10.1016/j.fm.2018.01.003. 29526204 PMC7127387

[jfds70527-bib-0002] Batziakas, K. G. , M. Talavera , M. Swaney‐Stueve , C. L. Rivard , and E. D. Pliakoni . 2019. “Descriptive Analysis and Consumer Acceptability of Locally and Commercially Grown Spinach.” Journal of Food Science 84, no. 8: 2261–2268. 10.1111/1750-3841.14710. 31313301

[jfds70527-bib-0056] Bedford, J. L. , and S. I. Barr . 2005. “Diets and selected lifestyle practices of self‐defined adult vegetarians from a population‐based sample suggest they are more 'health conscious'.” International Journal of Behavioral Nutrition and Physical Activity 2: 4. 10.1186/1479-5868-2-4. 15829014 PMC1090609

[jfds70527-bib-0003] Bellemare, M. F. , and N. Nguyen . 2018. “Farmers Markets and Food‐Borne Illness.” American Journal of Agricultural Economics 100, no. 3: 676–690. 10.1093/ajae/aay011.

[jfds70527-bib-0004] Bimal Sheth, U. , M. A. Haque , M. J. Jang , et al. 2025. “From Soil to Salad: Strategies for Reducing Foodborne Illness Outbreaks.” Food Science and Nutrition 13, no. 4521: e4521. 10.1002/fsn3.4521. 39803216 PMC11717025

[jfds70527-bib-0005] Bucholtz, S. 2020. “Urban. Suburban. Rural. How Do Households Describe Where They Live?” *PD&R Edge*. Published August 3. https://www.huduser.gov/portal/pdredge/pdr‐edge‐frm‐asst‐sec‐080320.html.

[jfds70527-bib-0006] Busch, G. , E. Bayer , A. Spiller , and S. Kühl . 2022. “Factory Farming? Public Perceptions of Farm Sizes and Sustainability in Animal Farming.” PLOS Sustainability and Transformation 1, no. 10: e0000032.

[jfds70527-bib-0007] Carstens, C. K. , J. K. Salazar , and C. Darkoh . 2019. “Multistate Outbreaks of Foodborne Illness in the United States Associated With Fresh Produce From 2010 to 2017.” Frontiers in Microbiology 10: 2667. 10.3389/fmicb.2019.02667. 31824454 PMC6883221

[jfds70527-bib-0008] Centers for Disease Control and Prevention . 2016. “2016 Outbreak of Listeria Infections Linked to Packaged Salads Produced at Springfield, Ohio Dole Processing Facility (Final Update).” Accessed December 23, 2024. https://archive.cdc.gov/#/details?q=https://www.cdc.gov/listeria/outbreaks/bagged‐salads‐01‐16/index.htmlandstart=0androws=10andurl=https://www.cdc.gov/listeria/outbreaks/bagged‐salads‐01‐16/index.html.

[jfds70527-bib-0009] Centers for Disease Control and Prevention . 2022. “List of Multistate Foodborne Outbreak Notices.” Accessed December 23, 2024. https://www.cdc.gov/foodborne‐outbreaks/active‐investigations/all‐foodborne‐outbreak‐notices.html.

[jfds70527-bib-0010] Chen, H. , J. K. Ellett , R. Phillips , and Y. Feng . 2021a. “Small‐Scale Produce Growers' Barriers and Motivators to Value‐Added Business: Food Safety and Beyond.” Food Control 130: 108192.

[jfds70527-bib-0011] Chen, H. , A. J. Kinchla , N. Richard , A. Shaw , and Y. Feng . 2021b. “Produce Growers' On‐Farm Food Safety Education: A Review.” Journal of Food Protection 84, no. 4: 704–716. 33270894 10.4315/JFP-20-320

[jfds70527-bib-0012] Chen, H. , E. Kontor‐Manu , H. Zhu , G. Cheng , and Y. Feng . 2024. “Evaluation of the Handling Practices and Risk Perceptions of Dried Wood Ear Mushrooms in Asian Restaurants in the United States.” Journal of Food Protection 87, no. 1: 100198. 38007093 10.1016/j.jfp.2023.100198

[jfds70527-bib-0013] Code of Federal Regulations . 2022. “Title 7 CFR Part 205 – National Organic Program.” Electronic Code of Federal Regulations. Accessed February 4, 2024. https://www.ecfr.gov/current/title‐7/part‐205.

[jfds70527-bib-0014] Crandall, P. G. , E. C. Friedly , M. Patton , et al. 2011. “Consumer Awareness of and Concerns About Food Safety at Three Arkansas Farmers' Markets.” Food Protection Trends 31, no. 3: 156–165. https://www.foodprotection.org/files/food‐protection‐trends/Mar‐11‐Rainey.pdf.

[jfds70527-bib-0015] Ellison, B. , J. C. Bernard , M. Michelle Paukett , and U. C. Toensmeyer . 2016. “The Influence of Retail Outlet and FSMA Information on Consumer Perceptions of and Willingness to Pay for Organic Grape Tomatoes.” Journal of Economic Psychology 55: 109–119. 10.1016/j.joep.2016.05.002.

[jfds70527-bib-0016] Fan, X. , M. I. Gómez , and P. S. Coles . 2019. “Willingness to Pay, Quality Perception, and Local Foods: The Case of Broccoli.” Agricultural and Resource Economics Review 48, no. 3: 414–432. 10.1017/age.2019.21.

[jfds70527-bib-0017] Food Safety News . 2017. “Salmonella Outbreak Linked to Wisconsin Farmers Market Peas.” Marler Clark. Accessed February 27, 2024. https://www.foodsafetynews.com/2017/08/salmonella‐outbreak‐linked‐to‐wisconsin‐farmers‐market‐peas/.

[jfds70527-bib-0018] Food Safety News . 2024. “Swedish Cryptosporidium Outbreak Traced to Kale.” News Desk. Accessed May 18, 2025. https://www.foodsafetynews.com/2024/12/swedish‐cryptosporidium‐outbreak‐traced‐to‐kale/.

[jfds70527-bib-0019] Fung, F. , H.‐S. Wang , and S. Menon . 2018. “Food Safety in the 21st Century.” Biomedical Journal 41, no. 2: 88–95. 10.1016/j.bj.2018.03.003. 29866604 PMC6138766

[jfds70527-bib-0020] Gardner, T. J. , C. Fitzgerald , C. Xavier , et al. 2011. “Outbreak of Campylobacteriosis Associated With Consumption of Raw Peas.” Clinical Infectious Diseases 53, no. 1: 26–32. 10.1093/cid/cir249. 21653299

[jfds70527-bib-0021] Gedikoğlu, H. , and A. Gedikoğlu . 2021. “Consumers' Awareness of and Willingness to Pay for HACCP‐Certified Lettuce in the United States: Regional Differences.” Food Control 130: 108263. 10.1016/j.foodcont.2021.108263.

[jfds70527-bib-0022] Goetz, G. n.d. “Did Deer Cause Oregon's Strawberry Outbreak?.” Food Safety News. Published August 2011. https://www.foodsafetynews.com/2011/08/epis‐pinpoint‐strawberries‐in‐or‐e‐coli‐outbreak/.

[jfds70527-bib-0023] Günden, C. , and T. Thomas . 2012. “Assessing Consumer Attitudes Towards Fresh Fruit and Vegetable Attributes.” Journal of Food, Agriculture and Environment 10, no. 2: 85–88. https://www.researchgate.net/publication/286951353_Assessing_consumer_attitudes_towards_fresh_fruit_and_vegetable_attributes.

[jfds70527-bib-0024] Henke, K. A. , T. Alter , M. G. Doherr , and R. Merle . 2021. “From Stable to Table: Determination of German Consumer Perceptions of the Role of Multiple Aspects of Poultry Production on Meat Quality and Safety.” Journal of Food Protection 84, no. 8: 1400–1410. 10.4315/JFP-20-491. 33793777

[jfds70527-bib-0025] Hooper, D. , J. Coughlan , and M. Mullen . 2008. “Structural Equation Modelling: Guidelines for Determining Model Fit.” Electronic Journal of Business Research Methods 6, no. 1: 53–60.

[jfds70527-bib-0026] Kaczmarek, M. , S. V. Avery , and I. Singleton . 2019. “Microbes Associated With Fresh Produce: Sources, Types and Methods to Reduce Spoilage and Contamination.” Advances in Applied Microbiology 107: 29–82. 10.1016/bs.aambs.2019.02.001. 31128748

[jfds70527-bib-0027] Khouryieh, M. , H. Khouryieh , J. K. Daday , and C. Shen . 2019. “Consumers' Perceptions of the Safety of Fresh Produce Sold at Farmers' Markets.” Food Control 105: 242–247. 10.1016/j.foodcont.2019.06.003.

[jfds70527-bib-0028] Lawrence, M. 2022. “Enhancing pepper production in the U.S.” Accessed May 18, 2025. https://www.nifa.usda.gov/about‐nifa/blogs/enhancing‐pepper‐production‐us.

[jfds70527-bib-0029] Leiner, D. J. 2019. “Too Fast, Too Straight, Too Weird: Non‐Reactive Indicators for Meaningless Data in Internet Surveys.” Survey Research Methods 13, no. 3: 229–248.

[jfds70527-bib-0030] Manyi‐Loh, C. E. , S. N. Mamphweli , E. L. Meyer , G. Makaka , M. Simon , and A. I. Okoh . 2016. “An Overview of the Control of Bacterial Pathogens in Cattle Manure.” International Journal of Environmental Research and Public Health 13, no. 9: 843. 10.3390/ijerph13090843. 27571092 PMC5036676

[jfds70527-bib-0031] Martinez, S. W. , and T. Park . 2021. Marketing Practices and Financial Performance of Local Food Producers: A Comparison of Beginning and Experienced Farmers . Economic Information Bulletin No. 225. U.S. Department of Agriculture, Economic Research Service. https://www.ers.usda.gov/webdocs/publications/101786/eib‐225.pdf?v=9927.8.

[jfds70527-bib-0032] Moser, R. , R. Raffaelli , and D. Thilmany‐McFadden . 2011. “Consumer Preferences for Fruit and Vegetables With Credence‐Based Attributes: A Review.” International Food and Agribusiness Management Review 14, no. 2: 121–142. 10.22004/ag.econ.103990.

[jfds70527-bib-0033] Nasheri, N. , A. Vester , and N. Petronella . 2019. “Foodborne Viral Outbreaks Associated With Frozen Produce.” Epidemiology and Infection 147: e291. 10.1017/S0950268819001791. 31625499 PMC6813648

[jfds70527-bib-0034] NBC News . 2024. “Vegetables and Herbs Sold at Walmart and Aldi Recalled Due to Possible Listeria Contamination.” Accessed May 18, 2025. https://www.nbcnews.com/health/health‐news/vegetables‐herbs‐sold‐walmart‐aldi‐recalled‐due‐possible‐listeria‐cont‐rcna163785.

[jfds70527-bib-0035] Neill, C. L. , and R. B. Holcomb . 2019. “Does a Food Safety Label Matter? Consumer Heterogeneity and Fresh Produce Risk Perceptions Under the Food Safety Modernization Act.” Food Policy 85: 7–14. 10.1016/j.foodpol.2019.04.001.

[jfds70527-bib-0036] Onozaka, Y. , G. Nurse , and D. T. Thilmany McFadden . 2010. “Local Food Consumers: How Motivations and Perceptions Translate to Buying Behavior.” Choices: The Magazine of Food, Farm and Resource Issues 25, no. 1: 1–6. https://www.choicesmagazine.org/UserFiles/file/article_109.pdf.

[jfds70527-bib-0037] Ortega, D. L. , H. H. Wang , L. Wu , and N. J. Olynk . 2011. “Modeling Heterogeneity in Consumer Preferences for Select Food Safety Attributes in China.” Food Policy 36, no. 2: 318–324. 10.1016/j.foodpol.2010.11.030.

[jfds70527-bib-0038] Painter, J. A. , R. M. Hoekstra , T. Ayers , et al. 2013. “Attribution of Foodborne Illnesses, Hospitalizations, and Deaths to Food Commodities by Using Outbreak Data, United States, 1998–2008.” Emerging Infectious Diseases 19, no. 3: 407–415. 10.3201/eid1903.111866. 23622497 PMC3647642

[jfds70527-bib-0039] Pan, F. , X. Li , J. Carabez , et al. 2015. “Cross‐Sectional Survey of Indicator and Pathogenic Bacteria on Vegetables Sold From Asian Vendors at Farmers' Markets in Northern California.” Journal of Food Protection 78, no. 3: 602–608. 10.4315/0362-028X.JFP-14-095. 25719888

[jfds70527-bib-0040] Paramithiotis, S. , E. H. Drosinos , and P. N. Skandamis . 2017. “Food Recalls and Warnings Due to the Presence of Foodborne Pathogens—A Focus on Fresh Fruits, Vegetables, Dairy and Eggs.” ScienceDirect 18: 71–75. 10.1016/j.cofs.2017.11.007.

[jfds70527-bib-0041] Parra, P. A. , H. Kim , M. A. Shapiro , R. B. Gravani , and S. D. Bradley . 2014. “Home Food Safety Knowledge, Risk Perception, and Practices Among Mexican‐Americans.” Food Control 37: 115–125.

[jfds70527-bib-0042] Pilař, L. , T. Balcarová , S. Rojik , I. Tichá , and J. Poláková . 2018. “Customer Experience With Farmers' Markets: What Hashtags Can Reveal.” International Food and Agribusiness Management Review 21, no. 6: 755–770. 10.22434/IFAMR2017.0039.

[jfds70527-bib-0043] Raison, B. , and J. C. Jones . 2020. “Virtual Farmers Markets: A Reflective Essay on a Rural Ohio Project.” Journal of Agriculture, Food Systems, and Community Development 9, no. 4: 1–12. 10.5304/jafscd.2020.094.020.

[jfds70527-bib-0044] Rakola, B. 2021. “USDA Celebrates National Farmers Market Week.” U.S. Department of Agriculture. Published July 29. https://www.usda.gov/media/blog/2021/07/29/usda‐celebrates‐national‐farmers‐market‐week.

[jfds70527-bib-0045] Scheinberg, J. A. , E. G. Dudley , J. Campbell , et al. 2017. “Prevalence and Phylogenetic Characterization of *Escherichia coli* and Hygiene Indicator Bacteria Isolated From Leafy Green Produce, Beef, and Pork Obtained From Farmers' Markets in Pennsylvania.” Journal of Food Protection 80, no. 2: 237–244. 10.4315/0362-028X.JFP-16-282. 28221988

[jfds70527-bib-0046] Stewart, H. , and D. Dong . 2018. The Relationship Between Patronizing Direct‐to‐Consumer Outlets and a Household's Demand for Fruits and Vegetables, Economic Research Report No. ERR‐242. USDA Economic Research Service. Economic Research Service. https://www.ers.usda.gov/publications/pub‐details/?pubid=86877.

[jfds70527-bib-0047] U.S. Food & Drug Administration . 2018. “Country Fresh Orlando LLC, Recalls Product Because of Possible Health Risk.” Accessed May 18, 2025. https://www.fda.gov/safety/recalls‐market‐withdrawals‐safety‐alerts/country‐fresh‐orlando‐llc‐recalls‐product‐because‐possible‐health‐risk.

[jfds70527-bib-0048] U.S. Food & Drug Administration . 2021. “Updated: Baker Farms Recalls Various Brand Name of Kale Due to Listeria monocytogenes Contamination.” Accessed May 18, 2025. https://www.fda.gov/safety/recalls‐market‐withdrawals‐safety‐alerts/updated‐baker‐farms‐recalls‐various‐brand‐name‐kale‐due‐listeria‐monocytogenes‐contamination.

[jfds70527-bib-0049] U.S. Food & Drug Administration . 2022. “Outbreak Investigation of *E. coli* O157:H7 – Spinach (November 2021).” Accessed May 18, 2025. https://www.fda.gov/food/outbreaks‐foodborne‐illness/outbreak‐investigation‐e‐coli‐o157h7‐spinach‐november‐2021.

[jfds70527-bib-0051] U.S. Food & Drug Administration . 2024a. “FSMA Final Rule on Produce Safety: Standards for the Growing, Harvesting, Packing, and Holding of Produce for Human Consumption.” Accessed February 4, 2024. https://www.fda.gov/food/food‐safety‐modernization‐act‐fsma/fsma‐final‐rule‐produce‐safety.

[jfds70527-bib-0052] U.S. Food & Drug Administration . 2024b. “Recalls, Market Withdrawals, and Safety Alerts.” Accessed May 26, 2025. https://www.fda.gov/safety/recalls‐market‐withdrawals‐safety‐alerts.

[jfds70527-bib-0053] Whitt, C. 2020. “A Look at America's Family Farms.” USDA Economic Research Service, Resource and Rural Economics Division. Published January 23. https://www.usda.gov/media/blog/2020/01/23/look‐americas‐family‐farms.

[jfds70527-bib-0054] Wu, W. , A. Zhang , R. D. van Klinken , P. Schrobback , and J. M. Muller . 2021. “Consumer Trust in Food and the Food System: A Critical Review.” Foods 10, no. 10: 2490. 34681539 10.3390/foods10102490 PMC8536093

[jfds70527-bib-0055] Yu, H. , Z. Lin , M. S. Lin , J. A. Neal , and S. A. Sirsat . 2022. “Consumers' Knowledge and Handling Practices Associated With Fresh‐Cut Produce in the United States.” Foods 11, no. 14: 2167. 10.3390/foods11142167. 35885411 PMC9318181

