# Supplementary material for: Consumers’ Food Safety Expectations and Risk Perceptions of Produce From Small and Medium‐Sized Farms
Source: J Food Sci. 2025 Sep 23;90(9):e70527. doi: 10.1111/1750-3841.70527 (PMC12457814; doi:10.1111/1750-3841.70527)
Supplement: Supplementary file 1 — Supplementary Materials: jfds70527‐sup‐0001‐Appendix.docx [file JFDS-90-0-s001.docx]

**Appendices:**

APPENDIX a. CONSUMERS produce SAFETY SURVEY

This study is focused on what consumers think about the safety of vegetables harvested from small- and medium-sized farms.

IRB Number 2020-1493

**Block Agreement**

1. [Description of the research]

- I agree
- I do not agree

**Block Quotas (Qualtrics)**

1. In which state do you currently reside?

- Drop list of states

1. Which gender do you identify the most with?

- Male
- Female
- Prefer not to say

1. What is your race and/or ethnicity? *[Select all that apply]*

- White non-Hispanic
- Hispanic or Latino
- Asian or Pacific Islander
- Native American
- African American
- Other, specify __________

1. What is your highest level of education?

- Less than High School Diploma/GED
- High School Diploma/GED
- Associate’s Degree
- Bachelor’s Degree
- Graduate Degree
- Prefer not to answer

1. Please estimate your total household income before taxes from 2020.

- Less than $25,000
- $25,000 – $49,999
- $50,000 - $74,999
- $75,000 - $99,999
- $100,000 - $149,999
- $150,000 - $199,000
- $200,000 and above

1. Please describe the area in which you live:

- Urban
- Suburban
- Rural

**Block 0 Screener**

1. Are you the primary shopper for yourself and/or your household?

- Yes
- No *[Skip to end]*

1. Have you purchased any of the following **fresh** produce in the past month: bell peppers, spinach, or kale (**NOT** canned, frozen, or in a ready-to-eat meal)?

- Yes
- No *[Skip to end]*

1. *(For those who met the criteria)* From the list of produce below, which of them have you purchased in the past month? *[Select all that apply]*

- Bell peppers
- Kale
- Spinach

**Block 1 Purchasing Practices and Handling Practices**

1. How would you define small- and medium-sized produce farms? *[Select all that apply]*

- Farms that have an average annual value of produce sold of $30,000 or less.
- Farms that have an average annual value of produce sold of $575,000 or less.
- Farms that have an average annual value of produce sold between $575,000 to $1M (one million).
- I use the production area to define small- and medium-sized farms. Please, specify the production area___________________
- I use the number of workers to define small- and medium-sized farms. Please, specify the number of workers __________________

1. *(Definition of small- and medium-sized farms) For this survey, we will define small-sized farms as those that have an average annual value of produce sold of $30,000 or less. Medium-sized farms will be those that have an average annual value of produce sold of $575,000 or less. And large-sized farms are those that exceed the previously mentioned amounts.*
2. Where do you normally buy your produce? *[Select all that apply]*

- Local supermarkets
- Specialized markets
- Farmers’ Market
- CSA (Community Supported Agriculture)
- Roadside stands
- Directly from the farms
- Online websites (e. g. Imperfect Foods, Misfit Market, Farmbox, Amazon Fresh)
- Online grocery delivery/Apps (e. g. Instacart, local supermarket app)
- None of the above, specify

1. *(According to answer Screener, Question 3)* How do you usually prepare your bell peppers? *[Select all that apply]*

- Boiling
- Steaming
- Blanching
- Roasting
- Stir-frying
- Grilling
- I eat them raw

1. *(According to answer Screener, Question 3)* How do you usually prepare your spinach? *[Select all that apply]*

- Boiling
- Steaming
- Blanching
- Roasting
- Stir-frying
- Grilling
- I eat them raw

1. *(According to answer Screener, Question 3)* How do you usually prepare your kale? *[Select all that apply]*

- Boiling
- Steaming
- Blanching
- Roasting
- Stir-frying
- Grilling
- I eat them raw

In the following section we want to know more about how you purchase and store produce. Please indicate how often you do each one of the next practices.

1. I make sure the bell peppers I purchase are firm and not bruised.

- Never
- Sometimes
- Always

1. I make sure the spinach and/or kale I purchase is not wilted or turning into a different color.

- Never
- Sometimes
- Always

1. I separate the vegetables from raw meat, chicken, or fish at the market, using individual bags.

- Never
- Sometimes
- Always

1. I typically pack a cooler, insulated bag, or ice packs when planning to purchase produce.

- Never
- Sometimes
- Always

1. I wash the produce I purchase if it is not in a container that says it was pre-washed.

- Never
- Sometimes
- Always

1. I store the produce I purchase inside the refrigerator.

- Never
- Sometimes
- Always

1. I store the vegetables in a separate part of the refrigerator from raw meat, chicken, or fish.

- Never
- Sometimes
- Always

**Block 2 Produce Food Safety Knowledge**

In the following section you are going to answer some questions regarding your knowledge of on-farm activities.

1. At the farm, soil and water can be the source of disease-causing microorganisms that can contaminate produce.

- True
- False
- I don’t know

1. Farmers can use raw manure as a soil amendment right before the harvest of leafy greens, like spinach.

- True
- False
- I don’t know

1. Damaged or bruised produce do not have a higher risk of causing foodborne illness if the produce is properly washed before consumption.

- True
- False
- I don’t know

1. On-farm food safety practices can help reduce the risk of the produce from carrying harmful bacteria.

- True
- False
- I don’t know

*[Extra questions]*

1. Do you consider food safety to be a **minimum quality standard** for your fresh produce?

For this survey, **the minimum quality standard** refers to a characteristic or condition that a fresh produce must have.

- Yes
- No

1. Should small- or medium- sized-farms have to follow the same food safety rules as larger farms?

- Yes
- No
- Not certain

**Block 3 Food Safety Risk Attitudes and Perceptions**

1. In the following section we want to know about your risk perception of certain situations.

Please, drag the bar to the best spot that corresponds to your opinion. A value of 0 means: “not at all willing to take risks” and a value of 10 means “very willing to take risks.”

| **General Risk Assessment** |
| --- |
| Are you generally a person who is fully prepared to take risks? |
| **Health Decisions** |
| When making a health decision, are you generally a person who is fully prepared to take health risks? |
| **Financial decision** |
| When making a financial decision, are you generally a person who is fully prepared to take financial risks? |
| **Eating behavior** |
| When choosing what to eat, are you generally a person who is fully prepared to make risky food choices? |
| When eating **spinach** (**that is NOT pre-washed** by the manufacturer and **will NOT be cooked**), are you generally a person who **will NOT wash** the **raw spinach** before consuming it? |
| When eating **kale** (**that is NOT pre-washed** by the manufacturer and **will NOT be cooked**), are you generally a person who **will NOT wash** the raw **kale** before consuming it? |
| When eating **bell peppers** (**that are NOT pre-washed** by the manufacturer and **will NOT be cooked**), are you generally a person who **will NOT wash** the raw **bell peppers** before consuming them? |
| **IMC question** |
| If you are paying attention, please drag the bar through value “5.” *[we can include them even though they do not select value “5”]* |

1. **(Attitudes)** In the following section we want to know how important some characteristics are for you when you assess the **food safety risk** of purchasing your produce. Rate each one of the characteristics according to your opinion from “Not at all important” to “Extremely important”

|  | **Not at all important** | **Slightly important** | **Moderately important** | **Very important** | **Extremely important** |
| --- | --- | --- | --- | --- | --- |
| **Hazards** | | | | | |
| Free of bacterial contamination |  |  |  |  |  |
| Free of harmful chemicals |  |  |  |  |  |
| Free of pesticide residues |  |  |  |  |  |
| **Location** | | | | | |
| Locally grown |  |  |  |  |  |
| Produce from small and medium-sized farms |  |  |  |  |  |
| **Labels** | | | | | |
| Pre-washed labels |  |  |  |  |  |
| Ready-to-eat labels |  |  |  |  |  |
| Safety inspected labels |  |  |  |  |  |
| Government certified traceability |  |  |  |  |  |

1. In your opinion, how large is the influence of the following groups along the food supply chain on **produce safety** (transmission or spread of harmful bacteria such as *E. coli* or *Salmonella*). Rate each one of the actors/stations from “Not at all influential” to “Extremely influential.”

|  | **Not at all influential** | **Slightly influential** | **Somewhat influential** | **Very influential** | **Extremely influential** |
| --- | --- | --- | --- | --- | --- |
| Government Agencies |  |  |  |  |  |
| Farmer |  |  |  |  |  |
| Produce Transportation |  |  |  |  |  |
| Produce packing facility |  |  |  |  |  |
| Retailer (e.g. Walmart, ALDI, Winn Dixie, Publix) |  |  |  |  |  |
| Chef/meal preparer |  |  |  |  |  |
| Me as a consumer |  |  |  |  |  |

1. Headline

Please, read the following outbreak headline and select who you would blame for this outbreak happening.

**“Multistate Outbreak of Shiga Toxin-producing *Escherichia coli* O157:H7 Infections Linked to Organic Spinach and Spring Mix Blend”**


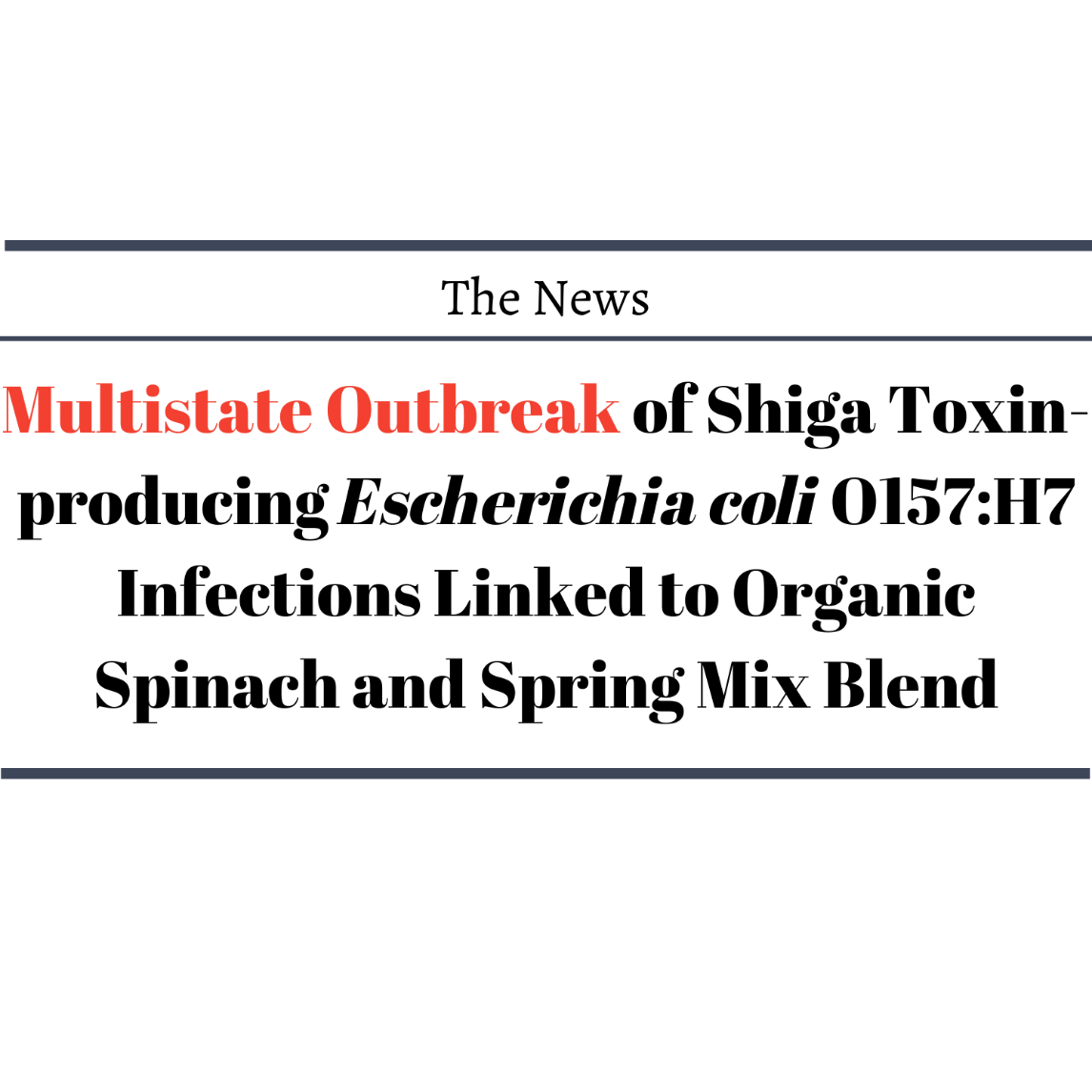


| Government Agencies |
| --- |
| Farmer |
| Produce Transportation |
| Produce packing facility |
| Retailer (e.g. Walmart, ALDI, Winn Dixie, Publix) |
| Chef/meal preparer |
| Me as a consumer |
| All of them |

1. Why would you blame XX? *[Open-ended question - Ask with the name of each actor/station according to the participant’s answer]*

**Block 5 Small and Medium Farms perception**

1. How important are the following attributes for you when purchasing produce? Rate each one of the characteristics according to your opinion from “Not at all important” to “Extremely important.”

|  | **Not at all important** | **Slightly important** | **Moderately important** | **Very important** | **Extremely important** |
| --- | --- | --- | --- | --- | --- |
| Price |  |  |  |  |  |
| Organic |  |  |  |  |  |
| Appearance |  |  |  |  |  |
| Locally grown |  |  |  |  |  |
| Fair treatment of Labor |  |  |  |  |  |
| Safety |  |  |  |  |  |

1. What are the differences between the produce purchase from **small and medium-sized farms vs.** **large farms**? [select all that apply]

- Higher quality
- Fresher
- Less environmental impact
- Safer to eat
- Help to sustain local farms
- Help for community growth
- More expensive
- Less expensive
- Benefits the economy
- None of the above
- All of the above

1. In the following section, we want to know how much you agree about purchasing produce directly from **small and medium-sized farms**. Rate each one of the statements according to your opinion from “Strongly disagree” to “Strongly agree.”

|  | **Strongly Disagree** | **Disagree** | **Neither Agree nor Disagree** | **Agree** | **Strongly Agree** |
| --- | --- | --- | --- | --- | --- |
| **Procurement** | | | | | |
| Small and medium-sized farms produce prices are very variable. |  |  |  |  |  |
| Delivery options from small and medium-sized farms are too limited. |  |  |  |  |  |
| Small and medium-sized farms do not offer a variety of produce. |  |  |  |  |  |
| Small and medium-sized farms only have seasonal produce (e. g. strawberries in summer, pumpkins in fall). |  |  |  |  |  |
| The quality of produce from small and medium-sized farms is variable (e. g. size, color). |  |  |  |  |  |
| Small and medium-sized farms do not offer precut, packaged, or other value-added processing options. |  |  |  |  |  |
| **Food availability** | | | | | |
| There are not close small or medium-sized farms in my area. |  |  |  |  |  |
| The market in which I purchase my produce do not sell produce from small and medium-sized farms. |  |  |  |  |  |

| **Food Safety** | | | | | |
| --- | --- | --- | --- | --- | --- |
| Small and medium-sized farms do not use adequate on-farm food safety practices. |  |  |  |  |  |
| On-farm food safety practices are not a priority for small and medium-sized farms. |  |  |  |  |  |
| Small and medium-sized farms follow few food safety practices. |  |  |  |  |  |

1. Headlines

Please, rate how much you are concerned after reading the following headlines.

1. **50 People Got Sick from Spinach Grown on a Small Farm.** [small-farm centric news]


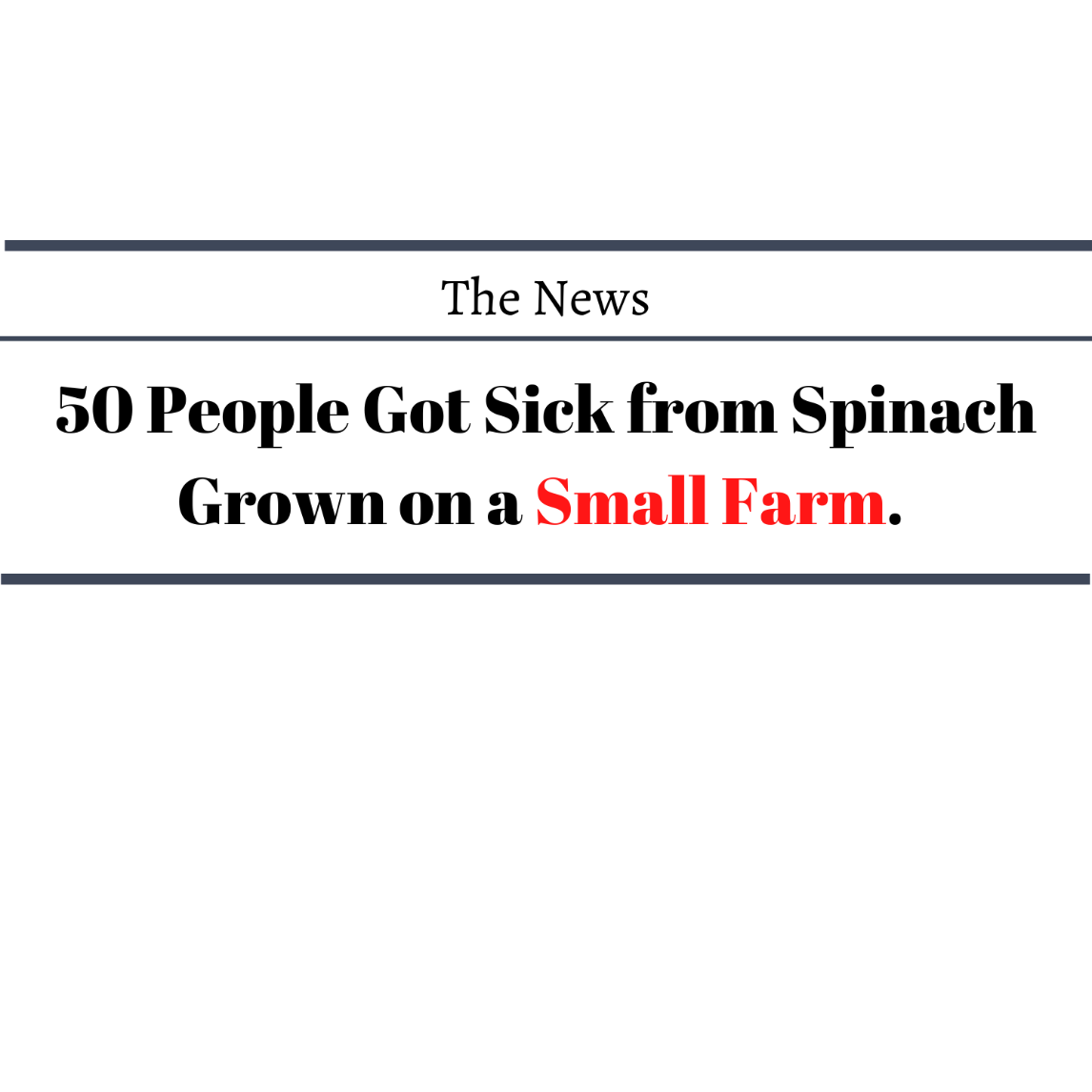


- (1) Not concerned at all
- (2) Somewhat concerned
- (3) Very concerned

1. **50 People Got Sick from Spinach Grown on a Large Farm.** [large-farm centric news]


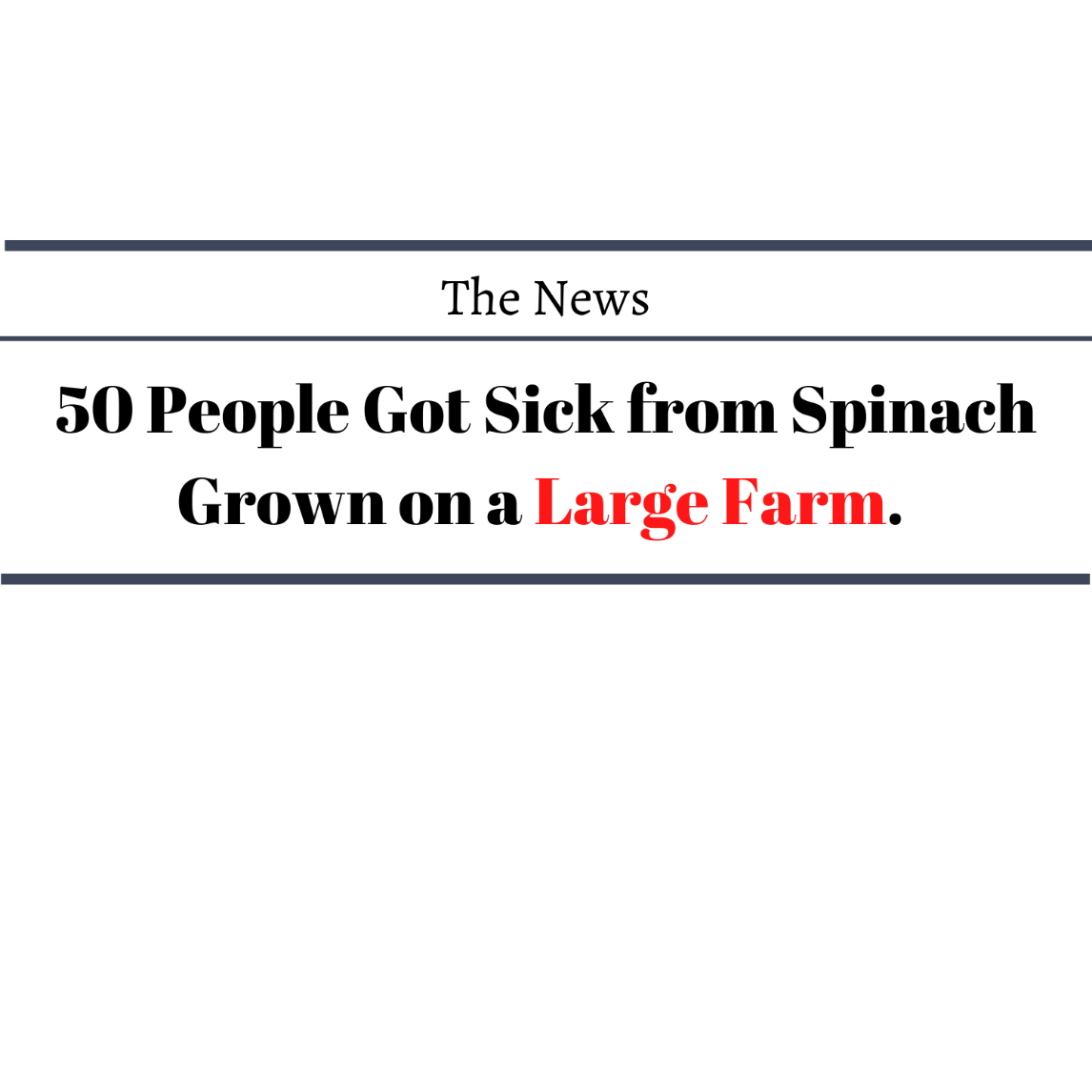


- (1) Not concerned at all
- (2) Somewhat concerned
- (3) Very concerned

**Produce Safety Rule**

*The Produce Safety Rule*

The Produce Safety rule establishes, for the first time, science-based minimum standards for the safe growing, harvesting, packing, and holding of fruits and vegetables grown for human consumption. This rule was implemented under the U.S. Food and Drug Administration (FDA).

However, not all farms are required to follow this rule. Some exemptions include small farms producing and selling fresh produce and whose annual value of produce sale is $30,000 or less.

- I have read the information (select to continue with the next section)

1. After reading the previous section, do you agree that small-sized farms should be exempt from the food safety regulation?

- Yes
- No
- Not certain, specify _______

1. Why do you agree that small-sized farms should be exempt from food safety regulation? *(if yes is selected)* *[Select all that apply]*

- They are operated by families.
- They benefit the community.
- Their production is small.
- We have to support local grown produce.
- Other, specify __________

1. Why do you disagree with the exemption of small-sized farms from the food safety regulation? *(if no is selected)* *[Select all that apply]*

- All farms should be regulated in the same way.
- The produce has to be safe for consumption.
- The food safety regulations are easy to follow.
- Other specify__________

1. If the produce you purchase follows the food safety regulation, would you like to see it on a food label?

- Yes
- No
- Not certain

**Block 9 Extra Demographic Characteristics.**

1. Do you live with children under the age of 18?

- Yes
- No

1. Do you live with children aged 5 or younger?

- Yes
- No

1. Do you live with adults aged 65 or above?

- Yes
- No

APPENDIX b. LOGISTIC REGRESSION ON THE LEVEL OF INFLUENCE OF DIFFERENT ACTORS ON THE PRODUCE SAFETY

Table Appendix B. Logistic regression regarding the level of influence of “Government Agencies” in produce Safety.

| **Sociodemographic** | **Likelihood Ratio test** (*P* value) | ***P* value** | **OR** | **95% CI** |
| --- | --- | --- | --- | --- |
| *Gender* | 0.305 |  |  |  |
| Female |  | 0.305 | 1.142 | (0.886; 1.471) |
| Male *(baseline)* |  | - | - | - |
| *Age range* | 0.007* |  |  |  |
| 18-24 |  | 0.005* | 0.498 | (0.305; 0.813) |
| 25-34 |  | 0.015* | 0.577 | (0.370; 0.899) |
| 35-44 |  | 0.056 | 0.648 | (0.415; 1.011) |
| 45-54 |  | 0.102 | 0.699 | (0.456; 1.074) |
| 55-64 |  | 0.469 | 1.178 | (0.756; 1.836) |
| 65 and above *(baseline)* |  | - | - | - |
| *Education level* | 0.005 * |  |  |  |
| Less than high school diploma/GED |  | 0.278 | 0.676 | (0.333; 1.372) |
| High school diploma/GED |  | <0.001* | 0.386 | (0.236, 0.632) |
| Some college (no degree) |  | 0.003* | 0.470 | (0.284; 0.778) |
| Associate’s degree |  | 0.029* | 0.519 | (0.288; 0.936) |
| Bachelor’s degree |  | 0.035* | 0.620 | (0.397; 0.968) |
| Graduate degree |  | - | - | - |
| *Household income* | 0.484 |  |  |  |
| Less than $25,000 |  | 0.429 | 0.776 | (0.414; 1.455) |
| $25,000 - $49,999 |  | 0.376 | 0.760 | (0.414; 1.395) |
| $50,000 - $74,999 |  | 0.276 | 0.714 | (0.389; 1.310) |
| $75,000 - $99,999 |  | 0.886 | 0.954 | (0.499; 1.825) |
| $100,000 - $149,999 |  | 0.080 | 0.586 | (0.322; 1.065) |
| $150,000 - $199,999 |  | 0.243 | 0.673 | (0.345; 1.310) |
| $200,000 and above (baseline) |  | - | - | - |
| *Area* | <0.001* |  |  |  |
| Rural |  | <0.001* | 0.456 | (0.314; 0.663) |
| Suburban |  | <0.001* | 0.518 | (0.382; 0.702) |
| Urban *(baseline)* |  |  |  |  |
| *Living with Children* | 0.983 |  |  |  |
| No children aged 5 or younger |  | 0.983 | 0.996 | (0.718; 1.384) |
| Children aged 5 or younger *(baseline)* |  | - | - | - |
| *Living with Elderly* | 0.574 |  |  |  |
| No adults aged 65 or above |  | 0.574 | 1.089 | (0.808; 1.469) |
| Adults aged 65 or above *(baseline)* |  | - | - | - |
| *Eating habits* | 0.327 |  |  |  |
| No vegetarians or vegans |  | 0.327 | 0.856 | (0.627; 1.168) |
| Vegetarians or vegans *(baseline)* |  | - | - | - |

**P ≤ 0.05*

*To interpret the results, the model was performed with and ascending parameter for the dependent variable.*


Table Appendix B. Logistic regression regarding the level of influence of “Produce Transportation” in produce Safety.

| **Sociodemographic** | **Likelihood Ratio test** (*P* value) | ***P* value** | **OR** | **95% CI** |
| --- | --- | --- | --- | --- |
| *Gender* | 0.354 |  |  |  |
| Female |  | 0.354 | 1.129 | (0.874; 1.458) |
| Male *(baseline)* |  | - | - | - |
| *Age range* | 0.404 |  |  |  |
| 18-24 |  | 0.179 | 1.407 | (0.855; 2.315) |
| 25-34 |  | 0.098 | 1.455 | (0.933; 2.269) |
| 35-44 |  | 0.035 | 1.624 | (1.034; 2.552) |
| 45-54 |  | 0.461 | 1.172 | (0.768; 1.789) |
| 55-64 |  | 0.282 | 1.275 | (0.819; 1.983) |
| 65 and above *(baseline)* |  | - | - | - |
| *Education level* | 0.437 |  |  |  |
| Less than high school diploma/GED |  | 0.863 | 0.941 | (0.468; 1.892) |
| High school diploma/GED |  | 0.089 | 0.659 | (0.408, 1.066) |
| Some college (no degree) |  | 0.372 | 0.798 | (0.487; 1.309) |
| Associate’s degree |  | 0.131 | 0.637 | (0.354; 1.143) |
| Bachelor’s degree |  | 0.211 | 0.756 | (0.488; 1.172) |
| Graduate degree *(baseline)* |  | - | - | - |
| *Household income* | 0.884 |  |  |  |
| Less than $25,000 |  | 0.485 | 1.247 | (0.672; 2.313) |
| $25,000 - $49,999 |  | 0.971 | 0.989 | (0.543; 1.800) |
| $50,000 - $74,999 |  | 0.536 | 1.205 | (0.667; 2.177) |
| $75,000 - $99,999 |  | 0.543 | 1.205 | (0.667; 2.177) |
| $100,000 - $149,999 |  | 0.859 | 1.055 | (0.586; 1.898) |
| $150,000 - $199,999 |  | 0.991 | 0.996 | (0.516; 1.923) |
| $200,000 and above *(baseline)* |  | - | - | - |
| *Area* | 0.033* |  |  |  |
| Rural |  | 0.019 | 0.639 | (0.440; 0.929) |
| Suburban |  | 0.021* | 0.697 | (0.513; 0.948) |
| Urban *(baseline)* |  | - | - | - |
| *Living with Children* | 0.648 |  |  |  |
| No children aged 5 or younger |  | 0.648 | 0.925 | (0.664; 1.291) |
| Children aged 5 or younger *(baseline)* |  | - | - | - |
| *Living with Elderly* | 0.143 |  |  |  |
| No adults aged 65 or above |  | 0.143 | 0.802 | (0.596; 1.078) |
| Adults aged 65 or above *(baseline)* |  | - | - | - |
| *Eating habits* | 0.224 |  |  |  |
| No vegetarians or vegans |  | 0.224 | 1.212 | (0.889; 1.654) |
| Vegetarians or vegans *(baseline)* |  | - | - | - |

**P ≤ 0.05*

*To interpret the results, the model was performed with and ascending parameter for the dependent variable.*

Table Appendix B. Logistic regression regarding the level of influence of “Produce Packing House” in produce Safety.

| **Sociodemographic** | **Likelihood Ratio test** (*P* value) | ***P* value** | **OR** | **95% CI** |
| --- | --- | --- | --- | --- |
| *Gender* | 0.271 |  |  |  |
| Female |  | 0.271 | 1.155 | (0.894; 1.491) |
| Male *(baseline)* |  | - | - | - |
| *Age range* | 0.015* |  |  |  |
| 18-24 |  | 0.008* | 0.509 | (0.309; 0.839) |
| 25-34 |  | 0.038* | 0.625 | (0.401; 0.975) |
| 35-44 |  | 0.412 | 0.827 | (0.525; 1.302) |
| 45-54 |  | 0.088 | 0.691 | (0.453; 1.056) |
| 55-64 |  | 0.547 | 1.147 | (0.734; 1.791) |
| 65 and above *(baseline)* |  | - | - | - |
| *Education level* | 0.231 |  |  |  |
| Less than high school diploma/GED |  | 0.871 | 1.063 | (0.508; 2.222) |
| High school diploma/GED |  | 0.043* | 0.604 | (0.371, 0.983) |
| Some college (no degree) |  | 0.197 | 0.721 | (0.438; 1.185) |
| Associate’s degree |  | 0.439 | 0.791 | (0.437; 1.432) |
| Bachelor’s degree |  | 0.417 | 0.833 | (0.535; 1.296) |
| Graduate degree *(baseline)* |  | - | - | - |
| *Household income* | 0.664 |  |  |  |
| Less than $25,000 |  | 0.279 | 1.412 | (0.756; 2.637) |
| $25,000 - $49,999 |  | 0.484 | 1.240 | (0.679; 2.263) |
| $50,000 - $74,999 |  | 0.271 | 1.402 | (0.768; 2.561) |
| $75,000 - $99,999 |  | 0.625 | 1.173 | (0.618; 2.230) |
| $100,000 - $149,999 |  | 0.352 | 1.326 | (0.732; 2.402) |
| $150,000 - $199,999 |  | 0.661 | 0.863 | (0.446; 1.668) |
| $200,000 and above *(baseline)* |  | - | - | - |
| *Area* | 0.087 |  |  |  |
| Rural |  | 0.420 | 0.856 | (0.586; 1.249) |
| Suburban |  | 0.032* | 0.713 | (0.523; 0.971) |
| Urban *(baseline)* |  |  |  |  |
| *Living with Children* | 0.127 |  |  |  |
| No children aged 5 or younger |  | 0.127 | 0.774 | (0.556; 1.076) |
| Children aged 5 or younger *(baseline)* |  | - | - | - |
| *Living with Elderly* | 0.965 |  |  |  |
| No adults aged 65 or above |  | 0.965 | 1.007 | (0.748; 1.354) |
| Adults aged 65 or above *(baseline)* |  | - | - | - |
| *Eating habits* | 0.505 |  |  |  |
| No vegetarians or vegans |  | 0.505 | 1.112 | (0.814; 1.519) |
| Vegetarians or vegans *(baseline)* |  | - | - | - |

**P ≤ 0.05*

*To interpret the results, the model was performed with and ascending parameter for the dependent variable.*

Table Appendix B. Logistic regression regarding the level of influence of “Retailer” in produce Safety.

| **Sociodemographic** | **Likelihood Ratio test** (*P* value) | ***P* value** | **OR** | **95% CI** |
| --- | --- | --- | --- | --- |
| *Gender* | 0.939 |  |  |  |
| Female |  | 0.939 | 0.990 | (0.768; 1.276) |
| Male *(baseline)* |  | - | - | - |
| *Age range* | 0.004* |  |  |  |
| 18-24 |  | 0.062 | 0.629 | (0.386; 1.023) |
| 25-34 |  | 0.007* | 0.546 | (0.352; 0.846) |
| 35-44 |  | 0.800 | 0.944 | (0.607; 1.470) |
| 45-54 |  | 0.099 | 0.701 | (0.460; 1.069) |
| 55-64 |  | 0.396 | 1.211 | (0.778; 1.884) |
| 65 and above *(baseline)* |  | - | - | - |
| *Education level* | 0.913 |  |  |  |
| Less than high school diploma/GED |  | 0.895 | 1.048 | (0.520; 2.112) |
| High school diploma/GED |  | 0.455 | 0.831 | (0.511, 1.350) |
| Some college (no degree) |  | 0.394 | 0.807 | (0.493; 1.322) |
| Associate’s degree |  | 0.515 | 0.824 | (0.461; 1.474) |
| Bachelor’s degree |  | 0.723 | 0.923 | (0.595; 1.434) |
| Graduate degree *(baseline)* |  | - | - | - |
| *Household income* | 0.132 |  |  |  |
| Less than $25,000 |  | 0.381 | 1.308 | (0.717; 2.388) |
|  |  |  |  |  |
| $25,000 - $49,999 |  | 0.852 | 0.946 | (0.529; 1.693) |
| $50,000 - $74,999 |  | 0.139 | 1.549 | (0.868; 2.763) |
| $75,000 - $99,999 |  | 0.927 | 0.971 | (0.519; 1.818) |
| $100,000 - $149,999 |  | 0.927 | 1.028 | (0.576; 1.834) |
| $150,000 - $199,999 |  | 0.940 | 0.975 | (0.509; 1.869) |
| $200,000 and above *(baseline)* |  | - | - | - |
| *Area* | 0.089 |  |  |  |
| Rural |  | 0.249 | 0.803 | (0.554; 1.166) |
| Suburban |  | 0.028* | 0.710 | (0.523; 0.964) |
| Urban *(baseline)* |  |  |  |  |
| *Living with Children* | 0.222 |  |  |  |
| No children aged 5 or younger |  | 0.222 | 0.816 | (0.588; 1.131) |
| Children aged 5 or younger *(baseline)* |  | - | - | - |
| *Living with Elderly* | 0.520 |  |  |  |
| No adults aged 65 or above |  | 0.520 | 1.101 | (0.821; 1.476) |
| Adults aged 65 or above *(baseline)* |  | - | - | - |
| *Eating habits* | 0.844 |  |  |  |
| No vegetarians or vegans |  | 0.844 | 1.031 | (0.758; 1.404) |
| Vegetarians or vegans *(baseline)* |  | - | - | - |

**P ≤ 0.05 To interpret the results, the model was performed with and ascending parameter for the dependent variable.*

Table Appendix B. Logistic regression regarding the level of influence of “Chef” in produce Safety.

| **Sociodemographic** | **Likelihood Ratio test** (*P* value) | ***P* value** | **OR** | **95% CI** |
| --- | --- | --- | --- | --- |
| *Gender* | 0.023* |  |  |  |
| Female |  | 0.023* | 1.347 | (1.042; 1.742) |
| Male *(baseline)* |  | - | - | - |
| *Age range* | <0.001* |  |  |  |
| 18-24 |  | <0.001* | 0.425 | (0.258; 0.698) |
| 25-34 |  | 0.001* | 0.475 | (0.303; 0.745) |
| 35-44 |  | 0.005* | 0.522 | (0.332; 0.823) |
| 45-54 |  | 0.045* | 0.643 | (0.418; 0.990) |
| 55-64 |  | 0.298 | 1.276 | (0.806; 2.017) |
| 65 and above *(baseline)* |  | - | - | - |
| *Education level* | 0.275 |  |  |  |
| Less than high school diploma/GED |  | 0.481 | 0.774 | (0.379; 1.580) |
| High school diploma/GED |  | 0.034* | 0.588 | (0.360, 0.960) |
| Some college (no degree) |  | 0.372 | 0.797 | (0.483; 1.313) |
| Associate’s degree |  | 0.131 | 0.637 | (0.355; 1.144) |
| Bachelor’s degree |  | 0.170 | 0.732 | (0.468; 1.143) |
| Graduate degree *(baseline)* |  | - | - | - |
| *Household income* | 0.325 |  |  |  |
| Less than $25,000 |  | 0.190 | 1.511 | (0.815; 2.802) |
| $25,000 - $49,999 |  | 0.351 | 1.327 | (0.732; 2.407) |
| $50,000 - $74,999 |  | 0.057 | 1.775 | (0.983; 3.205) |
| $75,000 - $99,999 |  | 0.718 | 1.125 | (0.593; 2.135) |
| $100,000 - $149,999 |  | 0.171 | 1.517 | (0.836; 2.753) |
| $150,000 - $199,999 |  | 0.566 | 1.208 | (0.634; 2.302) |
| $200,000 and above *(baseline)* |  | - | - | - |
| *Area* | 0.057 |  |  |  |
| Rural |  | 0.120 | 0.740 | (0.507; 1.081) |
| Suburban |  | 0.017* | 0.689 | (0.507; 0.936) |
| Urban *(baseline)* |  |  |  |  |
| *Living with Children* | 0.003* |  |  |  |
| No children aged 5 or younger |  | 0.003* | 0.609 | (0.436; 0.849) |
| Children aged 5 or younger *(baseline)* |  | - | - | - |
| *Living with Elderly* | 0.665 |  |  |  |
| No adults aged 65 or above |  | 0.665 | 0.937 | (0.696; 1.260) |
| Adults aged 65 or above *(baseline)* |  | - | - | - |
| *Eating habits* | 0.649 |  |  |  |
| No vegetarians or vegans |  | 0.649 | 1.075 | (0.788; 1.466) |
| Vegetarians or vegans *(baseline)* |  | - | - | - |

**P ≤ 0.05*

*To interpret the results, the model was performed with and ascending parameter for the dependent variable.*

Table Appendix B. Logistic regression regarding the level of influence of “Consumer” in produce Safety.

| **Sociodemographic** | **Likelihood Ratio test** (*P* value) | ***P* value** | **OR** | **95% CI** |
| --- | --- | --- | --- | --- |
| *Gender* | 0.040* |  |  |  |
| Female |  | 0.040* | 1.311 | (1.013; 1.697) |
| Male *(baseline)* |  | - | - | - |
| *Age range* | 0.012* |  |  |  |
| 18-24 |  | 0.052 | 0.611 | (0.371; 1.005) |
| 25-34 |  | 0.005* | 0.525 | (0.336; 0.820) |
| 35-44 |  | 0.076 | 0.665 | (0.424; 1.044) |
| 45-54 |  | 0.501 | 0.862 | (0.560; 1.327) |
| 55-64 |  | 0.530 | 1.159 | (0.732; 1.834) |
| 65 and above *(baseline)* |  | - | - | - |
| *Education level* | 0.842 |  |  |  |
| Less than high school diploma/GED |  | 0.727 | 0.879 | (0.428; 1.314) |
| High school diploma/GED |  | 0.378 | 0.800 | (0.487, 1.314) |
| Associate’s degree |  | 0.552 | 0.858 | (0.517; 1.422) |
| Bachelor’s degree |  | 0.261 | 0.713 | (0.395; 1.286) |
| Graduate degree |  | 0.244 | 0.765 | (0.487; 1.201) |
| Some college (no degree) *(baseline)* |  | - | - | - |
| *Household income* | 0.428 |  |  |  |
| Less than $25,000 |  | 0.231 | 1.461 | (0.785; 2.716) |
| $25,000 - $49,999 |  | 0.408 | 1.287 | (0.708; 2.340) |
| $50,000 - $74,999 |  | 0.052 | 1.802 | (0.994; 3.269) |
| $75,000 - $99,999 |  | 0.286 | 1.422 | (0.745; 2.713) |
| $100,000 - $149,999 |  | 0.123 | 1.596 | (0.881; 2.891) |
| $150,000 - $199,999 |  | 0.473 | 1.270 | (0.661; 2.443) |
| $200,000 and above *(baseline)* |  | - | - | - |
| *Area* | 0.047* |  |  |  |
| Rural |  | 0.075 | 0.708 | (0.485; 1.036) |
| Suburban |  | 0.015* | 0.682 | (0.500; 0.929) |
| Urban *(baseline)* |  |  |  |  |
| *Living with Children* | 0.573 |  |  |  |
| No children aged 5 or younger |  | 0.573 | 0.909 | (0.653; 1.266) |
| Children aged 5 or younger *(baseline)* |  | - | - | - |
| *Living with Elderly* | 0.448 |  |  |  |
| No adults aged 65 or above |  | 0.448 | 0.892 | (0.664; 1.198) |
| Adults aged 65 or above *(baseline)* |  | - | - | - |
| *Eating habits* | 0.283 |  |  |  |
| No vegetarians or vegans |  | 0.283 | 1.185 | (0.869; 1.616) |
| Vegetarians or vegans *(baseline)* |  | - | - | - |

**P ≤ 0.05*

*To interpret the results, the model was performed with and ascending parameter for the dependent variable.*
